# The long non-coding RNA NEAT1 contributes to aberrant STAT3 signaling in pancreatic cancer and is regulated by a metalloprotease-disintegrin ADAM8/miR-181a-5p axis

**DOI:** 10.1007/s13402-024-01001-0

**Published:** 2024-10-16

**Authors:** Yutong Gao, Kimia Zandieh, Kai Zhao, Natalia Khizanishvili, Pietro Di Fazio, Xiangdi Yu, Leon Schulte, Michelle Aillaud, Ho-Ryun Chung, Zachary Ball, Marion Meixner, Uta-Maria Bauer, Detlef Klaus Bartsch, Malte Buchholz, Matthias Lauth, Christopher Nimsky, Lena Cook, Jörg W. Bartsch

**Affiliations:** 1 Department of Neurosurgery, Philipps-University Marburg, Baldingerstrasse, 35043 Marburg, Germany; 2 Department of Visceral, Thoracic and Vascular Surgery, Philipps-University Marburg, Baldingerstrasse, 35033 Marburg, Germany; 3 Department of Anesthesiology, Guizhou Provincial People’s Hospital, The Affiliated Hospital of Guizhou University, Guiyang, Guizhou 550000 China; 4 Institute for Lung Research, Philipps-University Marburg, Hans-Meerwein-Strasse 2, 35043 Marburg, Germany; 5 Institute for Medical Bioinformatics and Biostatistics, Philipps-University Marburg, 35033 Marburg, Germany; 6 Department of Chemistry, Rice University, Houston, TX USA; 7 Institute for Molecular Biology and Tumor Research (IMT), Philipps-University Marburg, Marburg, Germany; 8 Department of Gastroenterology, Endocrinology, Metabolism and Infection, Center for Tumor and Immunology (ZTI), Philipps-University Marburg, Marburg, Germany

**Keywords:** PDAC, STAT3, ADAM8, LncRNA NEAT1, MiR-181a-5p, Ubiquitination, Proteasome

## Abstract

**Purpose:**

Pancreatic ductal adenocarcinoma (PDAC) is one of the most lethal cancers and several studies demonstrate that STAT3 has critical roles throughout the course of PDAC pathogenesis.

**Methods:**

TCGA, microarray, and immunohistochemistry data from a PDAC cohort were used for clinical analyses. Panc89 cells with ADAM8 knockout, re-expression of ADAM8 mutants, and Panc1 cells overexpressing ADAM8 were generated. Gene expression analyses of ADAM8, STAT3, long non-coding (lnc) RNA NEAT1, miR-181a-5p and ICAM1 were performed by quantitative PCR. Subcellular fractionation quantified NEAT1 expression in cytoplasm and nucleus of PDAC cell lines. Cell proliferation, scratch, and invasion assays were performed to detect growth rate, migration and invasion capabilities of cells. Gain and loss of function experiments were carried out to investigate the biological effects of lncRNA NEAT1 and miR-181a-5p on PDAC cells and downstream genes. Dual-luciferase reporter gene assay determined interaction and binding sites of miR-181a-5p in lncRNA NEAT1. Pull down assays, RNA binding protein immunoprecipitation (RIP), and ubiquitination assays explored the molecular interaction between lncRNA NEAT1 and STAT3.

**Results:**

High ADAM8 expression causes aberrant STAT3 signaling in PDAC cells and is positively correlated with NEAT1 expression. NEAT1 binding to STAT3 was confirmed and prevents STAT3 degradation in the proteasome as increased degradation of STAT3 was observed in ADAM8 knockout cells and cells treated with bortezomib. Furthermore, miRNA-181a-5p regulates NEAT1 expression by direct binding to the NEAT1 promoter.

**Conclusion:**

ADAM8 regulates intracellular STAT3 levels via miR-181a-5p and NEAT1 in pancreatic cancer.

**Supplementary Information:**

The online version contains supplementary material available at 10.1007/s13402-024-01001-0.

## Background

Pancreatic cancer deaths account for approximately 5% of various types of cancer [1]. It can be categorized into pancreatic ductal adenocarcinoma (PDAC), which accounts for approximately 85% of all cases, and pancreatic neuroendocrine tumors, accounting for 5% of all remaining cases [2]. PDAC is detected only in stage III (often unresectable, probable expansion to nearby blood vessels or nerves without metastasis to distant sites) or stage IV (metastatic, likely spread to other organs) patients; the 1-year survival rate is reported to reach a maximum of 20%, and the 5-year survival rate is around 10% [3]. The majority of pancreatic tumors strongly express A disintegrin and metalloproteinase (ADAM) 8 (ADAM8). ADAM8 is a type I transmembrane (TM) glycoprotein consisting of an N-terminal prodomain, followed by a metalloproteinase-, disintegrin (DIS)-, cysteine-rich-, epidermal growth factor (EGF)-like- and transmembrane (TM)-domain and a cytoplasmic tail [4]. Based on clinical data, a strong correlation between high ADAM8 expression levels and poor prognosis was demonstrated [5]. It was postulated that the enhanced proteolytic activity of ADAM8 may result in increased cell migration, invasion, and metastasis in PDAC [5]. However, these numerous effects of ADAM8 in tumor cells do not rely solely on its extracellular protease function. Rather, it is likely that the ADAM8 cytoplasmic domain could exert a prominent role for PDAC tumor progression. With regard to intracellular pathways, ADAM8 can activate focal adhesion kinase (FAK), extracellular regulated kinase (ERK1/2), and AKT/PI3 kinase signaling, which critically depend on the cytoplasmic domain [6–8]. As shown in glioblastoma cells, ADAM8 mediates angiogenesis by inducing the expression of osteopontin (SPP1) via signal transducer and activator of transcription 3 (STAT3) signaling [9]. Based on the diverse functions of ADAM8 in intracellular signaling, it was hypothesized that ADAM8 can mediate these functions through regulation of non-coding RNAs. Indeed, our previous study on glioblastoma revealed that ADAM8 alters the expression profile of the microRNA miR-181a-5p, thereby contributing to tumor aggressiveness [10].

Among non-coding RNAs, microRNAs (miRNAs) are non-coding single-stranded small RNAs of 21–23 nucleotides and regulate gene expression post-transcriptionally either through mRNA degradation or translational repression. In particular, miR-181a-5p inhibits cell migration, invasion and proliferation by repressing the ERK-matrix metalloprotease (MMP) pathway via targeting the Dual specificity mitogen-activated protein kinase kinase 1 (MAP2K1/MEK1) as shown in esophageal squamous cell carcinoma [11]. MiR-181a-5p also increases the sensitivity of HS578T breast cancer cells to cisplatin by inducing vitamin D receptor-mediated autophagy [12]. In addition, ADAM8 plays a crucial role in regulating miR-181a-5p expression as it downregulates expression of miR-181a-5p via activation of STAT3 and mitogen-activated protein kinase (MAPK) signaling, suggesting an ADAM8-dependent repression of miR-181a-5p [10]. There are numerous miRNA‒long non-coding RNA (lncRNA) interactions as important regulators of protein-coding genes in cancer. For instance, lncRNAs can compete with miRNAs for binding to the 3′-UTR of target mRNAs, leading to inhibition of miRNAs and activation of target proteins via a process termed sponging. Conversely, miRNAs can directly target lncRNAs and decrease their expression in cancer cells [13]. For example, miR-124-3p is markedly decreased in ovarian cancer patients, and its tumor suppressive role might be due to its regulation of the lncRNA “Nuclear enriched abundant transcript 1” (NEAT1) expression [14].

LncRNA NEAT1 is transcribed from the *MEN1* (multiple endocrine neoplasia locus 1) gene locus. It has been reported to exert an important role by modulating proliferation, apoptosis, migration and invasion in several cancers [15, 16]. However, the detailed underlying mechanisms have not been fully elucidated yet. NEAT1 has five known splice variants, and the two most abundant isoforms are NEAT1_1 and NEAT1_2, with transcript lengths of 3.735 and 22.741 nt, respectively. Together, these two isoforms of NEAT1 and certain RNA binding proteins constitute a pivotal nuclear structure named paraspeckles, with NEAT1_2 playing a major role in the formation of paraspeckles [17]. Whereas NEAT1_1 is not a key constituent of paraspeckles, it has been detected in several non-paraspeckle loci. This localization pattern implies paraspeckle-independent roles for NEAT1_1 [18]. A study revealed that several proteins can directly interact with NEAT1 but are not essential for the structure of paraspeckles [19]. Previous studies have also shown that NEAT1 expression is significantly upregulated during the progression of pancreatic cancer [20–23]. NEAT1 mediates the Zinc finger E-box-binding homeobox 2 (ZEB2) mRNA expression by sponging miR-506-3p to promote the epithelial-to-mesenchymal transition [22]. It can also cause STAT3 signaling by direct interaction, leading to CD4+ T-cell differentiation [24]. However, the functional link between ADAM8 and NEAT1 in PDAC needs to be further investigated. In the present study, we explored the mechanism by which ADAM8 modulates intracellular STAT3 signaling in a complex network involving miR-181a-5p and NEAT1. Importantly, STAT3 was identified as an interacting partner of NEAT1 that maintains aberrant STAT3 signaling in PDAC cells by preventing STAT3 degradation in the proteasome.

## Methods

### PDAC patient cohort

A total of 128 patients with PDAC who underwent surgical resection in the Department of Visceral Surgery at the University Hospital Marburg were enrolled in our study (see Table 1). All tumors were histologically staged by an experienced pathologist according to UICC-TNM (Union for International Cancer Control; tumor, node, metastasis) classification 2017 [15]. Samples were obtained from the tumor bank of the Department of Pathology and Department of Visceral, Thoracic, and Vascular Surgery at Marburg University. Ethical approval was granted by the local ethics committee at Marburg University, Faculty of Medicine (File Nr. 5/03). All patients provided written consent prior to participating in this study.

**Table 1:** Clinical data on the PDAC cohort used in this study for NGS analyses (mRNA expression) and for immunohistochemistry (protein)

Variables		NGS analysis (*n* = 78)	IHC staining (*n* = 50)
Gender	Males (%)	40 (51%)	24 (48%)
	Females (%)	38 (49%)	26 (52%)
Median age at surgery, years (range)		68 (47–85)	68 (47–85)
UICC Stage, number of patients (%)	I	11 (14%)	3 (6%)
	II	12 (15%)	19 (38%)
	III	49 (63%)	27 (54%)
	IV	6 (8%)	1 (2%)
Median survival, months (range)		22 (1–92)	23 (1–92)
Location	Head	69 (88%)	45 (90%)
	Body or tail	9 (12%)	5 (10%)

*Abbreviations UICC* Union for International Cancer Control

### Cell culture

Panc89 cells were cultured in RPMI-1640 (Gibco^TM^, Life Technologies) supplemented with 10% (v/v) fetal bovine serum (FBS) (Sigma‒Aldrich, Dreieich, Germany), 0.1 mg/mL penicillin‒streptomycin (Gibco^TM^, Life Technologies) and 1 mM sodium pyruvate (Gibco^TM^, Life Technologies). Panc1, HPAC, CFPAC, PaTu8988T, S007, MiaPaCa and BxPC3 cells were cultured in DMEM (Gibco^TM^, Life Technologies) supplemented with 10% (v/v) FBS (Sigma‒Aldrich), 0.1 mg/mL penicillin‒streptomycin (Gibco^TM^, Life Technologies) and 1 mM sodium pyruvate (Gibco^TM^, Life Technologies). All cell lines were cultivated in a humidified incubator at 37 °C and 5% CO_2_, and routinely tested for mycoplasma contamination.

### MiR-181a-5p mimic or inhibitor transfection

Lipofectamine™ RNAiMAX (Invitrogen) was used for transient transfection according to the manufacturer’s instructions. Cells were seeded in 6-well plates at a density of 500,000 cells/well and transfected with 0.01 μM hsa-miR-181a-5p mimic (miScript, Qiagen, Hilden, Germany) or inhibitor (miRCURY, Qiagen) after cell attachment. ON-TARGET plus nontargeting control pool (0.01 μM; Horizon Discovery, Cambridge, UK) was used as control miRNA. After 48 h, the cells were harvested, and miRNA expression was analyzed by RT‒qPCR. The sequence of the miR-181a-5p mimic was 5’-AACAUUCAACGCUGUCGGUGAGU’ 3’, and the sequence of the miR-181a-5p antisense (inhibitor) was 5’-ACTCACCGACAGCGTTGAATG’ 3’.

### Transient transfection for NEAT1 knockdown

NEAT1 knockdown in cells was performed utilizing Lipofectamine RNAiMAX (Invitrogen) according to the manufacturer’s instructions. Cells were seeded in 6-well plates at a density of 400,000 cells/well and transfected with 0.02 μM silencer select NEAT1 siRNA (Thermo Fisher Scientific, Dreieich, Germany) after attachment. ON-TARGET plus nontargeting control pool (0.01 μM; Horizon Discovery, Cambridge, UK) was used as the control siRNA. After 24 h, cells were harvested, and lncRNA expression was analyzed by RT‒qPCR.

### Transient transfection for NEAT1 overexpression

NEAT1 overexpression in cells was performed utilizing Lipofectamine LTX & PLUS Reagent (Invitrogen) according to the manufacturer’s instructions. Cells were seeded in 6-well plates at a density of 400,000 cells/well. The pcDNA3.1NEAT1 expression vector was cloned from a 3.7 kb NEAT1 fragment in pCRII_Topo (Addgene, USA) into the pcDNA3.1(-) vector by GenScript (Rijswijk, Netherlands). Cells were transfected with either 2.5 μg of pcDNA3.1-NEAT1 or with the pcDNA3.1 empty vector. After 48 h, cells were harvested, and lncRNA expression was analyzed by RT‒qPCR.

### Reverse transcription-quantitative polymerase chain reaction (RT‒qPCR)

Total miRNA from cells was isolated using the miRNeasy Tissue/Cells Advanced Mini Kit (Qiagen, Hilden, Germany) according to the manufacturer’s instructions. A MiRCURY LNA RT Kit (Qiagen) was used for reverse transcription, and a miRCURY LNA SYBR® Green PCR Kit was used for quantifying miRNA expression according to the manufacturer’s instructions. The YP00203_U6 snRNA miRCURY LNA PCR Assay (Qiagen) and miRCURY miRNA Assay hsa-miR-181a-5p (Qiagen) were used for the quantification of relative miR-181a-5p expression. For total mRNA and lncRNA isolation, Qiazol (Qiagen) was used, and then 2 μg of isolated RNA was transcribed into cDNA using an EcoDry^TM^ kit (Takara Bio, Inc.). RT‒qPCR was performed with iTaqTM Universal SYBR Green Supermix (Bio-Rad Laboratories GmbH, Göttingen, Germany). QuantiTect Primer Assay (Qiagen) of forward and reverse primers were diluted 1:10 in a total reaction volume of 20 μl using a StepOnePlus Real-time PCR system (ABI, Thermo Fisher Scientific, USA). The primers XS13 (ribosomal *RPLP0* gene) (fw: 5’-TGG GCA AGA ACA CCA TGA TG-3’; rev: 5’-AGT TTC TCC AGA GCT GGG TTG T-3’) were used for internal normalization of gene expression, and relative gene expression was calculated by the 2-ΔΔCt method.

### Protein extraction and Western blot

Cells were lysed using RIPA buffer (50 mM HEPES pH 7.4; 150 mM 542 NaCl; 1% (v/v) NP-40; 0.5% (w/v) Natriumdeoxycholate; 0.1% (w/v) SDS; 10 mM phenantrolin; 10 mM EDTA) supplemented with protease inhibitor-cocktail (A32955, Thermo Scientific, Waltham, MA, USA), phosphatase inhibitor mix (A32957, Thermo Scientific, USA), and incubated on ice for 30 min. After centrifugation at 12,000 × g for 15 min at 4 °C, the protein concentration was determined using the Pierce™ BCA Protein Assay Kit (Thermo Scientific). Then, proteins were boiled at 95 °C for 5 min. The extracted proteins were separated by SDS‒PAGE on 10% gels and then transferred to nitrocellulose membranes (GE Healthcare, Düsseldorf, Germany) using wet transfer. The membranes were blocked with 5% (w/v) milk powder (MP) in TBST for 1 h and subsequently incubated with the following primary antibodies at 4 °C overnight: anti-ADAM8 (PA5-47047, Thermo Fisher Scientific, 1:1000), anti-β-Tubulin (NB600-936, 1:2000; Novus Biological, Wiesbaden, Germany), anti-STAT3 (9139S, 1:1000; Cell Signaling Technology, Leiden, The Netherlands), and anti-pSTAT3 (Tyr705) (9145S, Cell Signaling Technology, 1:1000). After washing with TBST three times, membranes were incubated with horseradish peroxidase-conjugated secondary antibodies for 1 h at room temperature (1:4000; Abcam, Cambridge, UK). The membranes were washed three times with TBST and signals were detected using the Chemiluminescent HRP Substrate, Western Bright Sirius (Advansta, Hessisch Oldendorf, Germany) and the ChemiDoc MP Imaging System (Bio-Rad Laboratories GmbH). Signals were quantified using Image J.

### Cell proliferation assay

A total of 5000 cells were seeded in each well of a 96-well plate overnight for attachment. A CellTiter-Glo 3D cell viability assay (Promega, Walldorf, Germany) was performed to detect cell proliferation at 24, 48 and 72 h. Then, 50 μL of CellTiter-Glo 3D reagent was added to each well and mixed by shaking for 15 minutes. After incubation at room temperature in the dark for 15 min, luminescence was measured with a microplate reader (FLUOstar OPTIMA Microplate Reader, BMG Labtech, Offenburg, Germany).

### Scratch assay

A total of 50,000 cells in 70 μL of medium containing 10% FBS (v/v) were seeded per well of a Culture-Insert Well (Ibidi GmbH, Gräfelfing, Germany) in a 24-well plate overnight for attachment. Cells were starved overnight with medium supplemented with only 1% FBS. The medium was exchanged by fresh medium supplemented with 10% FBS on the next day and inserts were removed to start the migration assay. Images of each gap were taken at 0 and 10 h. Images were analyzed by Image J software.

### Cell invasion assay

Invasion assays were performed using ThinCert^TM^ Cell Culture Inserts with 8 μm pore diameters (Greiner Bio-One GmbH, Frickenhausen, Germany). The upper side of the Thincert was coated with 50 μL of Culture UltiMatrix RGF BME (Bio-Techne GmbH, Wiesbaden, Germany), which was diluted 1:2 with cold medium (without FBS) before seeding the cells. After solidifying for 1 h at 37 °C, 25,000 cells in 50 μL of medium containing 0.5% (v/v) FBS were seeded on the lower side of the Thincert. The cells were cultivated in an incubator for 5 h for attachment. Then, 750 μL of medium supplemented with 0.5% FBS was added to each well, and 250 μL of medium supplemented with 20% FBS was added to overlay the matrix. After 24 h, 4% (w/v) PFA was used to fix the cells for 30 min, and 0.3% Triton was used to permeabilize the cells for 30 min. The cells were subsequently stained with Hoechst 33,342 dye overnight for quantification. The Z-stack of five random viewing fields were recorded. For quantification, cells on the lower part of the Thincert (noninvasive cells) and cells in the matrix (invasive cells) were counted.

### Lumit immunoassay

A total of 25,000 cells were seeded per well in a 96-well plate overnight for attachment. After that, the medium was replaced with 40 μL of fresh cell medium. Next, 10 μL of lysis solution (Promega) was added to each well, followed by vigorous shaking of the plate for 20 min. Then, 50 μL of antibody mixture (anti-STAT3 and anti-pSTAT3, Cell Signaling Technology; Lagit anti-mouse antibody-LagBit, and Lumit anti-rabbit antibody SmBit, Promega) was added to each well, and the plate was gently shaken for 2 min. The plate was then incubated at room temperature for 90 min. Before luminescence was measured, 25 μL of Lumit detection reagent (Promega) was added and the plate was gently shaken for 2 min.

### Dual‑luciferase reporter gene assay

DNA sequences from NEAT1 containing either wild-type or mutant miR-181a-5p binding sites were synthesized and cloned via XhoI and NotI into psiCHECK2 to generate the corresponding reporter constructs (Fig. 4G). Panc89 cells were cotransfected with 0.4 μg of reporter construct and 0.01 μM miR-181a-5p mimic or control RNA (siCtrl). After 48 h, the cells were harvested and analyzed using a dual luciferase assay (Promega) according to the manufacturer’s instructions. All transfection assays were carried out in triplicate.

### NEAT1-protein interaction assay

Biotinylated NEAT1_1 or antisense NEAT1_1 was constructed by Biosense Bioscience Co., Ltd. (Guangzhou, China), and then incubated with cellular protein lysates from Panc89 cells. Then, 300 ml of streptavidin beads were added. Recovered proteins associated with NEAT1_1 or antisense NEAT1_1 were detected by western blotting.

### RNA binding protein immunoprecipitation (RIP) assay

RIP experiments were performed using the Magna Nuclear RIP™ kit (Millipore). Briefly, 3 × 10^7^ cells were prepared for lysis. Magnetic bead-antibody complexes for immunoprecipitation were prepared by using 10 μg of STAT3 antibody (Cell Signaling Technology). Then, RNA binding protein‒RNA complexes (RBPs) were immunoprecipitated according to the manufacturer’s instructions. Fold enrichment was calculated based on the 2-ΔΔCt method.

### Subcellular fractionation

Cells were collected as described and lysed using cytosolic lysis buffer supplemented with 5% NP-40 and 2 mM vanadyl ribonucleoside complex (VRC). After centrifugation at 1000 × g, the pellet was used to extract the nuclear proteins whereas the supernatant was used for extraction of cytoplasmic proteins. The supernatant was centrifuged at 13,000 × g, 1/10 of the total cytoplasmic cell lysate was used for protein detection, and 9/10 of the total cytoplasmic cell lysate for RNA extraction. Next, the pellet was treated with nuclear extraction buffer with 2 mM VRC added and centrifuged at 13,000 × g for 30 min. About 1/10 of the total nuclear cell lysate was used for protein detection and 9/10 of the total nuclear cell lysate for RNA extraction to verify enrichment of nuclear (Histone H3) and cytoplasmic proteins (GAPDH). Furthermore, qPCR analysis for nuclear RNA was performed with primers for U6 spliceosomal nuclear RNA and cytoplasmic RNA was detected using primers for ß-Actin. Enrichment for these RNAs was at least 5000× fold calculating the ratios of cytoplasmic over nuclear RNA for ß-Actin and at least 700× fold for U6 when calculating the ratio of nuclear over cytoplasmic RNA.

### Ubiquitination assay and bortezomib treatment

Ubiquitination assays were conducted using a Signal Seeker™ Ubiquitination Detection Kit (Cytoskeleton Inc., Denver, CO, USA). Briefly, cells were used from siRNA transfection experiments or were incubated with or without 1 ng/mL bortezomib for 16 hours prior to attachment and then harvested. Next, cell lysates were prepared using BlastR^TM^ Rapid Lysate Prep provided by this kit. After that, the protein concentration was detected. 1 mg of total protein was used for immunoprecipitation using Ubiquitination Affinity Beads and incubated on a rotating platform at 4 °C for two hours. About 20 μl of the lysate was saved as western input lysate control. Then the beads were collected by centrifugation at 3–5000 × g and washed three times. After the final wash and complete removal of buffer supernatant, 30 μl of Bead Elution Buffer was added. The beads were resuspended and incubated at room temperature for five minutes. Next, the bead suspension was gently transferred to the spin column and centrifuged at 9–10,000 × g for one minute at room temperature. Both of input lysate and IP lysate were denatured at 95 °C for five minutes followed by western blot. Primary antibodies of STAT3 and pSTAT3 were used as described above. This ubiquitination assay kit detected both of mono- and poly-ubiquitinated protein, so after ubiquitination precipitation and western blot, the displayed bands were all representative for ubiquitinated STAT3.

### Statistical analysis

For statistical analyses of normal distributed values, Student’s *t test*, for non-normal distribution, Mann-Whitney U test (two groups comparison) and for more than 2 group comparisons, a Kruskal-Wallis test was used. Data were considered not significant (*p* > 0.05, no asterisk), significant * (*p* < 0.05), highly significant ** (*p* < 0.01), or very highly significant *** (*p* < 0.001) and are expressed as the mean ± S.D.

## Results

### ADAM8 affects STAT3 signaling in PDAC cell lines

**Fig. 1:**
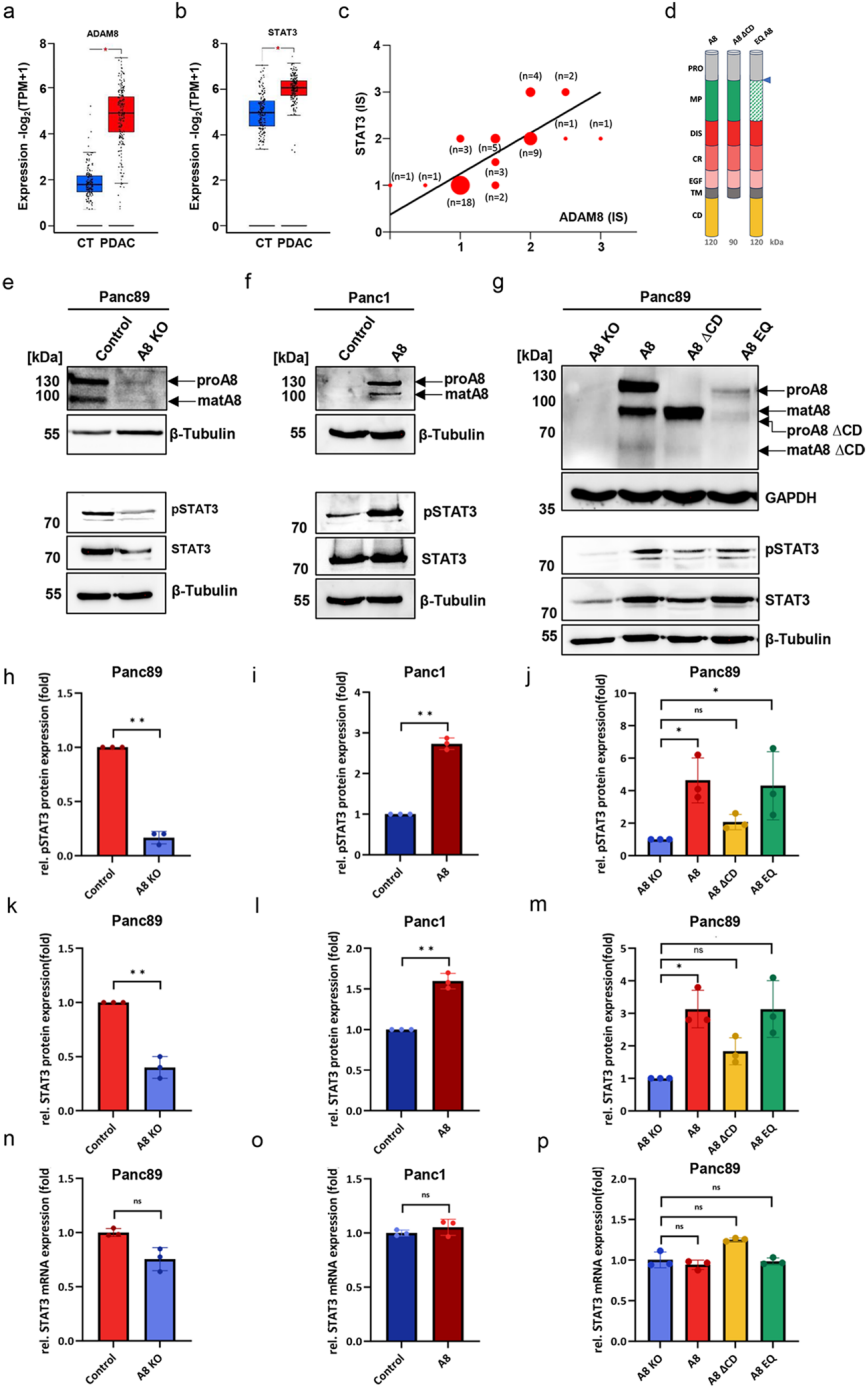
ADAM8 affects STAT3 signaling in PDAC cell lines. **a** and **b** Expression levels of ADAM8 and STAT3 in control (CT) and pancreatic ductal adenocarcinoma (PDAC) tissue taken from the GEPIA 2 database (matched TCGA normal and GTEx data). **c** Based on immunohistochemistry data from some of the Marburg PDAC patients (*n* = 50, see Table1), STAT3 expression correlated positively with ADAM8 expression in PDAC tumor tissue (*r* = 0.7564 and *p* value < 0.0001); **d** Schematic representation of the various domains of the human ADAM8 protein and the different domain variants with A8 with molecular weights below: ADAM8 (120 kDa); A8ΔCD: ADAM8 lacking the cytoplasmic domain (≈90 kDa); A8EQ: proform ADAM8 with an inactive metalloprotease domain (120 kDa); for all proforms, prodomain cleavage occurs (marked by blue arrow) to gain the mature ADAM8 protein. **e** After ADAM8 knockout, both STAT3 and pSTAT3 protein levels were decreased in Panc89_A8KO cells (*n* = 3). **f** ADAM8 overexpression resulted in increased STAT3 and pSTAT3 protein levels in Panc1 cells (*n* = 3). **g** STAT3 and pSTAT3 protein expression in Panc89 A8ΔCD cells was not restored as much as that in Panc89_A8 cells and Panc89_A8EQ cells (*n* = 3). **h** and **k** Quantitative results from Western blots are shown in (**e**), *n* = 3. **i** and **l** Quantitative results of (**f**), *n* = 3. **j** and **m** Quantitative results of (**g**), *n* = 3. **n**‒**p** Effect of ADAM8 expression on STAT3 mRNA levels in Panc89 cells (**n**) and Panc1 cells (**o**) and effect of re-expressing ADAM8 domain mutants on STAT3 mRNA expression in Panc89 cells (**p**). The data are presented as mean values ± S.D. Statistically significant differences were determined using Mann Whitney or Kruskal-Wallis test (* *p* < 0.05; ** p < 0.01)

Clinical data based on TCGA data (http://gepia2.cancer-pku.cn) show a significant upregulation of ADAM8 and STAT3 transcripts in PDAC tissues compared to healthy pancreas tissues (Fig. 1a, b). Furthermore, immunohistochemistry data from 50 patients of the Marburg PDAC cohort revealed a significant correlation between ADAM8 and STAT3 protein levels, with *r* = 0.7564 and *p* < 0.001 (Fig. 1c), but not for their respective transcripts (*r* = −0.0028; *p* = 0.97, data not shown). ADAM8, a metalloprotease-disintegrin, contains an active protease domain with several nonexclusive substrates for ectodomain shedding and extracellular matrix (ECM) protein cleavage. To address the function of the protease and the cytoplasmic domain, Panc89 cells with ADAM8 knockout (_A8KO) was constructed firstly and then a set of re-expression cell clones from Panc89_A8KO cells was generated, expressing either wild-type ADAM8 with an active protease (_A8), the protease-inactive variant ADAM8EQ (_A8EQ) with amino acid exchange E to Q at aa position 335, and an ADAM8 construct lacking the cytoplasmic domain (_A8ΔCD, Fig. 1d). Several cell clones were obtained and validated for ADAM8 expression by western blot (data not shown). For each domain variant, one representative Panc89 cell clone was used for further analyses (Fig. 1e, f and g). Concomitantly, the protein levels of total STAT3 and p-STAT3 were analyzed and found to correlate with the protein levels of ADAM8, suggesting that ADAM8 affects protein levels of STAT3 and p-STAT3 in PDAC cells (Fig. 1h, i, k and l), while STAT3 transcription is not affected by ADAM8 expression (Fig. 1n, o and p). These results were confirmed by Lumit® luminescence assays in Panc89 and Panc1 cells (Fig. S1). Moreover, when the domain variants of ADAM8 stably re-expressed in Panc89_A8KO cells were analyzed, wild-type ADAM8 (active protease; _A8) and protease-deficient ADAM8 (_A8EQ) were both able to increase STAT3/p-STAT3 levels, whereas the ADAM8 variant lacking the cytoplasmic domain (_A8ΔCD) was not able to significantly increase STAT3/p-STAT3 levels (Fig. 1g, j and m), suggesting that the cytoplasmic domain of ADAM8 is required to mediate the observed effects on STAT3 protein concentration and activation.

### ADAM8 expression is correlated with lncRNA NEAT1 expression in PDAC cell lines

**Fig. 2:**
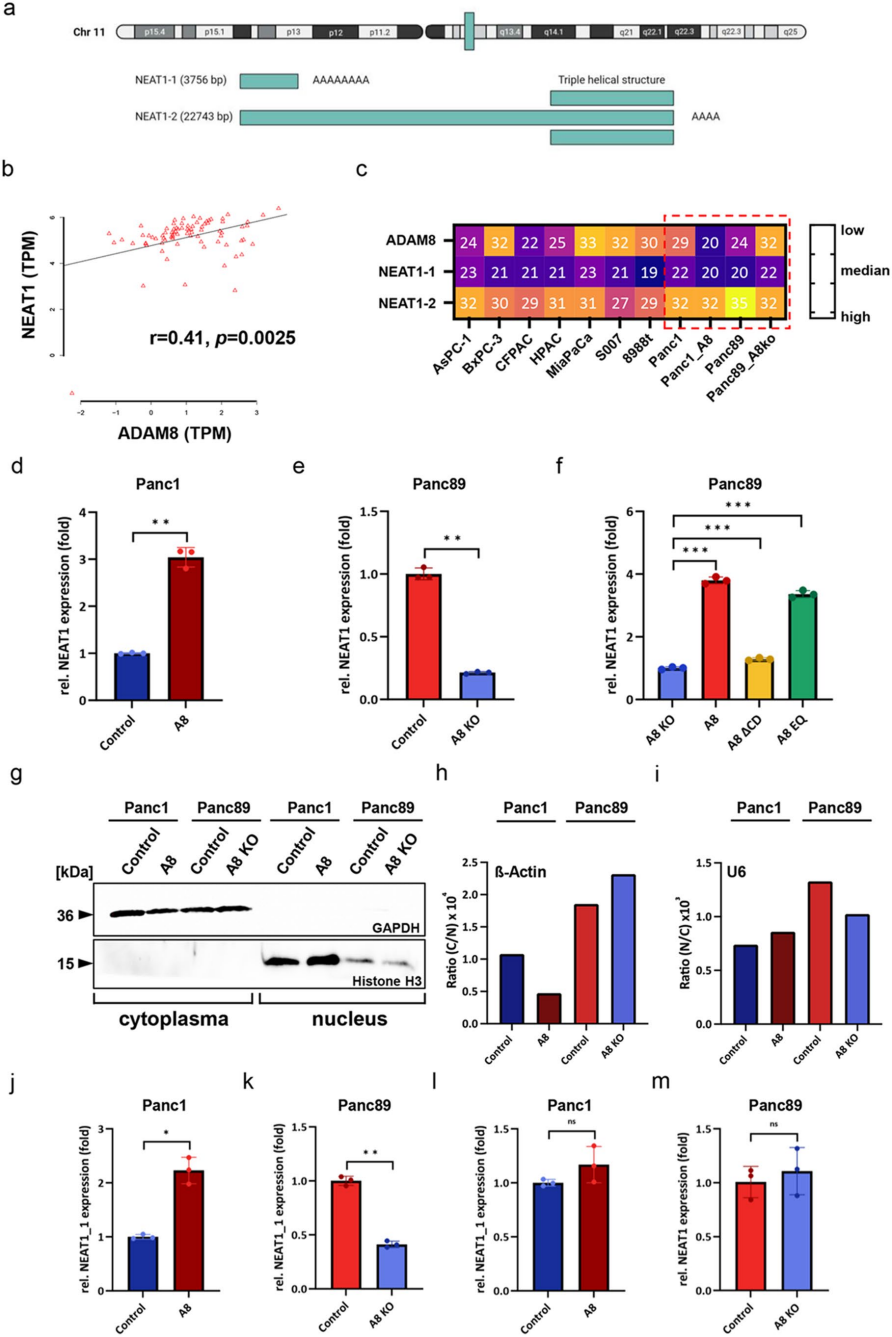
ADAM8 expression is correlated with lncRNA NEAT1 expression in PDAC cell lines. **a** Genomic location of NEAT1 on chromosome 11q13.1 (*upper line*) and organization of transcripts for either NEAT1_1 (3.7 kB transcript) or NEAT1_2 (22 kB transcript), both of which are transcribed from the same MEN1 gene locus. NEAT1_2 contains a unique triple helical structure at the 3′ end. **b** NEAT1 expression correlates positively with ADAM8 expression in the Marburg PDAC cohort (*n* = 78), with r = 0.41, *p* = 0.0025. **c** Mean Ct values of ADAM8, and the two transcripts for NEAT1_1 and NEAT1_2 in nine different PDAC cell lines, AsPC-1, BxPC-3, CFPAC,HPAC, MiaPaCa, S007, 8988t, Panc1 and Panc89, including Panc89_A8KO (ADAM8 knockout) and Panc1_A8 (ADAM8 overexpression), from the lowest Ct value of 35 for NEAT1_2 in Panc89 (*yellow*) to the highest Ct value of 20 (*purple*) in Panc1_A8. The four cell lines used on this study are boxed by a dotted red line (–) NEAT1 expression in Panc1_control and Panc1 ADAM8-overexpressing cells (**d**), in Panc89_control and Panc89_A8KO cells (**e**), and in Panc89_A8KO cells with re-expression of wild-type ADAM8, ADAM8∆CD, or ADAM8EQ with an inactive protease domain (**f**). Note that NEAT1 expression levels in Panc89_A8∆CD cells are similar to those in Panc89_A8KO cells. **g** Confirmation of successful subcellular fractionation of Panc1, Panc1_A8, Panc89, and Panc89_A8KO cells by western blot; GAPDH was used as a marker for the cytoplasm, histone H3 for the cell nucleus. **h** and **i** Confirmation of successful subcellular fractionation by qRT-PCR, the ratio of cytoplasmic and nuclear ß-Actin expression in these four cell lines (**h**), the ratio of nuclear and cytoplasmic U6 expression in these four cell lines (**i**); ß-Actin was used as marker for cytoplasm and U6 for nucleus. **j** Cytoplasmic NEAT1_1 expression in Panc1_control and Panc1_A8 cells. **k** Cytoplasmic NEAT1_1 expression in Panc89_control and Panc89_A8KO cells. **l** Nuclear NEAT1_1 expression in Panc1_control and Panc1_A8 cells. **m** Nuclear NEAT1_1 expression in Panc89_control and Panc89_A8KO cells. Data are presented as mean values ± S.D and statistical significance was determined by Mann-Whitney (2 groups) or Kruskal-Wallis (>2 groups) tests, respectively (*p < 0.05; **p < 0.01, ***p < 0.001)

Numerous related studies have demonstrated that interactions between lncRNAs and microRNAs play a key role in tumorigenesis [13, 14, 20, 22, 23]. For instance, lncRNAs can sponge microRNAs, leading to decreased microRNA expression, and microRNAs can also reversely regulate lncRNA expression. We found that the long non-coding RNA NEAT1, encoded by the *MEN1* gene locus (Fig. 2a), is positively correlated with ADAM8 expression in PDAC tissues from 78 patients of the Marburg cohort (Table1, Fig. 2b). NEAT1 has two transcript variants: a short transcript, NEAT1_1, 3.756 bp in length, which is often dysregulated in tumors, and a long transcript, NEAT1_2, 22.743 bp in length, which is important for the formation of paraspeckles (Fig. 2a). We quantified the expression levels of both transcripts, NEAT1_1 and NEAT1_2, in eleven commonly used PDAC cell lines. Although the expression levels of NEAT1_2 hardly changed in PDAC cells, we observed a tendency toward increased NEAT1_1 expression in those cell lines with elevated ADAM8 expression (Fig. 2c), particularly obvious in the last 4 cell lines of Panc89 and Panc1 in which the ADAM8 level is controlled by loss- and gain-of function. Furthermore, we explored the interaction between NEAT1 and ADAM8 by *ADAM8* knockout or *ADAM8* overexpression in PDAC cells and even in PDAC cells harboring different ADAM8 variants. In RT-qPCR assays, although the primers of NEAT1_1 was used, the expression levels of both isoforms were detected because NEAT1_1 sequence is part of NEAT1_2 sequence. The results showed that NEAT1 was upregulated after ADAM8 overexpression in Panc1 cells and downregulated after ADAM8 knockout in Panc89 cells compared to that in control cells (Fig. 2d and e). This was not observed for NEAT1_2 (Fig. S2). NEAT1 was also upregulated in both the Panc89_A8 and Panc89_A8EQ cell lines compared to the Panc89_A8KO cell line, but NEAT1 expression in the Panc89_A8ΔCD cell line was not upregulated as much as the expression in the ADAM8-expressing cell line and was rather comparable to the expression in the Panc89_A8KO cell line (Fig. 2f). This indicates that the cytoplasmic domain of ADAM8 strongly affects the expression of NEAT1. Although NEAT1 is an important element of paraspeckles, it can be translocated through the nuclear pore complex facilitated by a specific exportin and thereby enter the cytoplasm. To explore the intracellular distribution of NEAT1 and its dependence on ADAM8 expression in PDAC cells, nuclear and cytoplasmic NEAT1 were isolated separately and quantified by qPCR. Successful cellular fractionation was confirmed by western blotting using either a marker for cytoplasm (GAPDH) or the cell nucleus (histone H3, Fig. 2g). These results suggest that the concentrations of NEAT1_1 and NEAT1_2 in the cell nucleus were not affected by ADAM8 expression levels (Figs. 1g and S2). However, for cytoplasmic NEAT1 levels, only NEAT1_1 was upregulated after ADAM8 overexpression in Panc1 cells and downregulated in Panc89_A8KO cells (Fig. 2g).

### ADAM8 regulates miR-181a-5p expression in PDAC cell lines

**Fig. 3:**
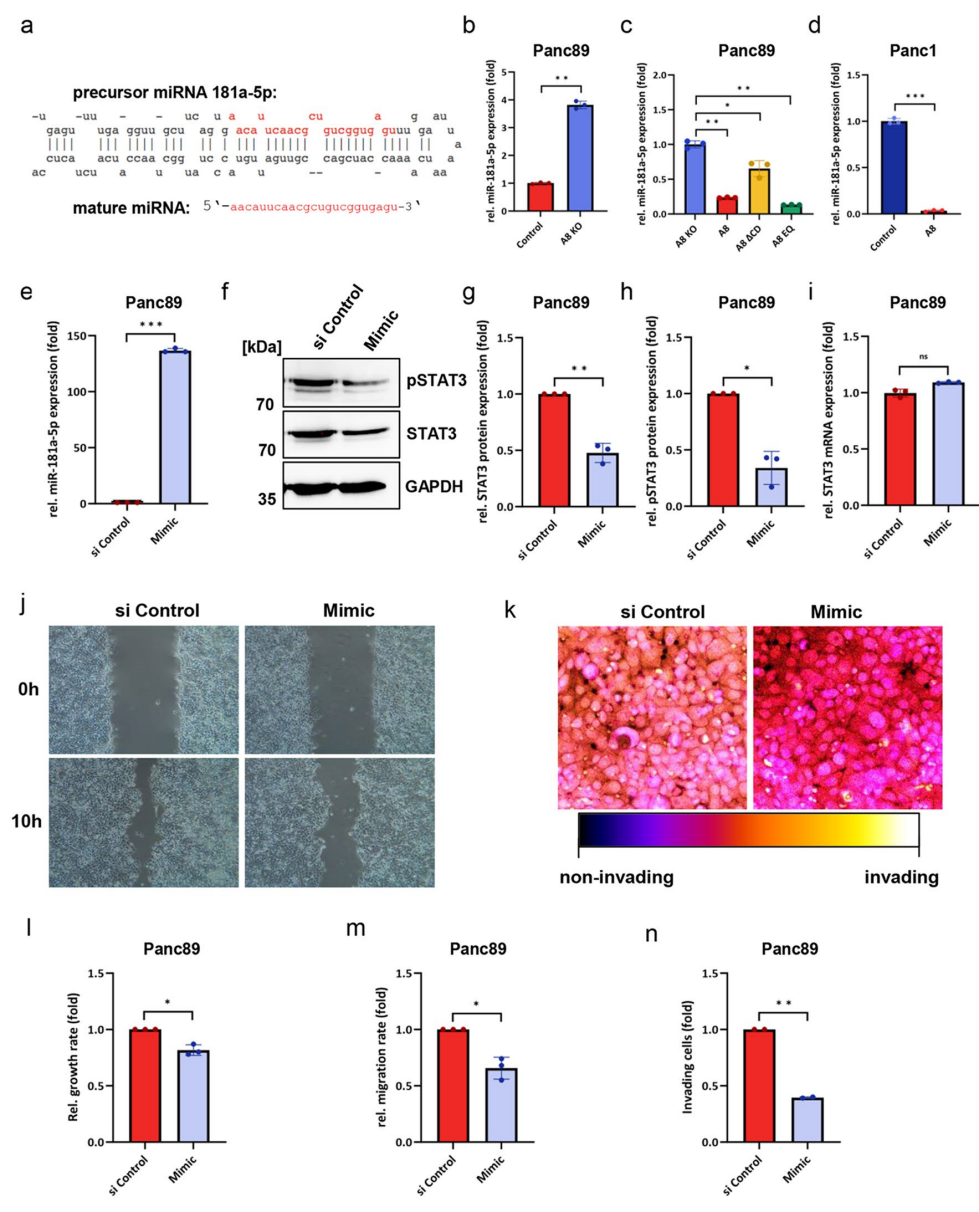
ADAM8 regulates miR-181a-5p expression in PDAC cell lines, affecting proliferation, migration and invasion behavior; **a** nucleotide sequence of miRNA181a-5p as precursor and mature miRNA; **b** MiR-181a-5p expression was upregulated after ADAM8 knockout in Panc89 cells. **c** MiR-181a-5p expression in Panc89_A8KO cells, Panc89_A8 cells, Panc89_A8ΔCD cells, and Panc89_A8EQ cells. **d** MiR-181a-5p expression was downregulated in Panc1 cells overexpressing ADAM8. **e** MiR-181a-5p expression levels after transfection of Panc89 cells with the miR-181a-5p mimic and **f** Determination of STAT3 and pSTAT3 protein levels after miR-181a-5p mimic overexpression (*n* = 3). **g** and **h**, Quantification of (**f**); **i** Effect of miR-181a-5p overexpression on STAT3 mRNA levels in Panc89 cells. **j** Scratch assays of Panc89 cells transfected with or without miR-181a-5p mimics. Images were acquired at 0 and 10 hours (*n* = 3). **k** Invasion assays of Panc89 cells transfected with or without miR-181a-5p mimic using transwell inserts (24 h) (*n* = 2). **l** Relative growth rate of Panc89 cells transfected with or without miR-181a-5p mimics after 48 h (*n* = 3). **m** Quantitative results of (**j**). **n** Quantitative result of (**k**). Data are presented as mean values ± S.D and Student’s *t test* was used with *p < 0.05; **p < 0.01; ***p < 0.001

Previous studies in glioblastoma cells have demonstrated ADAM8-dependent negative regulation of the 23-mer miRNA-181a-5p (Sequence in Fig. 3a [10]). To investigate this regulation in PDAC cells, Panc89 and Panc1 cells were analyzed for miRNA-181a-5p expression after knockout, re-expression of ADAM8 domain variants in Panc89 cells and overexpression in Panc1 cells (Fig. 3b–d). In both cell lines, expression levels of miRNA-181a-5p correlate with endogenous ADAM8 levels (ΔCt values of 19.7 in Panc1 and 26.4 in Panc89). Further, qPCR results showed that miRNA-181a-5p expression was upregulated after ADAM8 knockout and downregulated after ADAM8 overexpression compared to that in the respective control cells. Moreover, for ADAM8 domain variants, miRNA-181a-5p was downregulated in both the Panc89_A8 and Panc89_A8EQ cell lines compared to that in the Panc89_A8KO cell line, whereas miRNA-181a-5p expression in the Panc89_A8ΔCD cell line was not downregulated and was more comparable to that in the Panc89_A8KO cell line (Fig. 3c). We concluded that the ADAM8 cytoplasmic domain affects suppression of miRNA-181a-5p by ADAM8 in PDAC cells. Due to the presence of SH3 domains in the ADAM8 cytoplasmic domain, we further hypothesized that the effect on miRNA-181a-5p could be mediated by the tyrosine kinase Src, as ADAM8 is able to bind Src in hepatocarcinoma cells [25]. To test this possibility, a Src kinase inhibitor, PP2, was used at concentrations of 1 and 10 μM in Panc89 and Panc1 cells (Fig. S5). A slight but not significant increase in miRNA-181a-5p levels was observed only in Panc89 cells. This observation is in contrast to the effects observed in Panc89 and Panc1 cells with an SH3 inhibitor, an aminoquinoline-Rhodium(II) conjugate, compound 4B [26], which strongly increased miRNA-181a-5p expression levels in both cell lines (Fig. S5). suggesting that an SH3 binding protein is responsible for the regulation of miRNA-181a-5p that may be different from Src kinase. To investigate the effect of miRNA-181a-5p on PDAC cells in more detail, we transfected Panc89 cells with a miRNA-181a-5p mimic. The expression levels were approximately 135-fold greater than those in the control-transfected cells (Fig. 3e). Concomitantly, STAT3/p-STAT3 levels were significantly reduced by at least 50% (Fig. 3g and h), whereas the mRNA levels of STAT3 remained unchanged (Fig. 3i). Consistent with more aberrant STAT3 signaling in control cells than in mimic-transfected cells, the ability of these cells to proliferate, migrate, and invade was analyzed in more detail (Fig. 3j–n). Proliferation and migration of Panc89 cells were significantly (*p* < 0.01) reduced after mimic transfection; however, the strongest effect of miRNA-181a-5p mimic was observed on invasion (Fig. 3 m and n, *p* < 0.001), suggesting that miRNA181a-5p contributes to the invasive capacity of PDAC cells.

### MiRNA-181a-5p regulates NEAT1 expression by direct binding to NEAT1

**Fig. 4:**
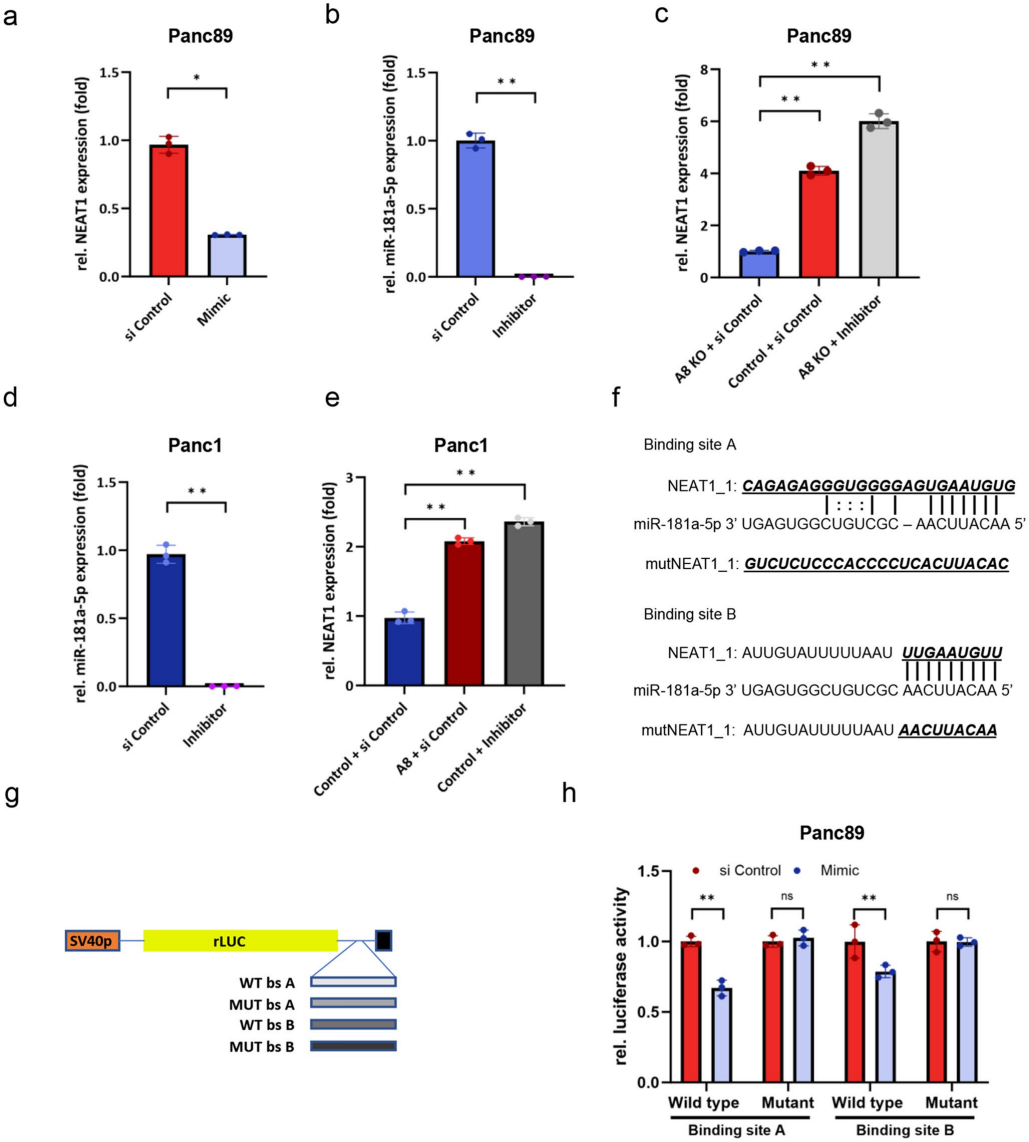
MiRNA-181a-5p regulates NEAT1 expression by direct binding to NEAT1. **a** NEAT1 expression levels in Panc89 cells after transfection with a miR-181a-5p mimic. **b** Confirmation of miR-181a-5p inhibition in Panc89_A8KO cells using an antisense miR-181a-5p (inhibitor). **c** NEAT1 levels were decreased after ADAM8 knockout in Panc89_A8KO cells but restored after miR-181a-5p inhibition. **d** Confirmation of miR-181a-5p inhibition in Panc1 cells. **e** NEAT1 expression in Panc1 cells was lower than that in Panc1_A8 cells but was upregulated after miR-181a-5p inhibition in Panc1 cells. **f** Nucleotide sequences of consensus and mutated binding sites A and B in the sequence of human NEAT1_1 for miR-181a-5p. **g** Cloning of the psiCHECK2 vector for analysis of miR-181a-5p binding sites A and B in the NEAT1 sequence. **h** Constructs containing NEAT1 sequences A and B in their wild-type and mutated forms, respectively, were transfected into Panc89 cells, and the relative luciferase activities of the indicated NEAT1_1 reporter construct were determined by dual luciferase assay (*n* = 3). For each construct, the siRNA control values were set to 1 and data are presented as mean values ± S.D and Student’s *t test* was used with *p < 0.05; **p < 0.01)

Since we observe opposed correlations between miRNA-181a-5p and NEAT1 with ADAM8 expression, respectively, we hypothesized that there could be a negative regulation of NEAT1 by miRNA-181a-5p. To investigate this, transfection of the miRNA-181a-5p mimic into Panc89 cells was performed and significantly reduced NEAT1 expression levels were observed (Fig. 4a). We further used antisense miRNA-181a-5p to suppress miRNA-181a-5p expression in Panc89_A8KO and Panc1 cell lines (Fig. 4b and d). Under these conditions, NEAT1 expression was significantly increased by 6-fold in Panc89 (Fig. 4c) and 2.3-fold in Panc1 cells (Fig. 4e). These expression levels were comparable to the NEAT1 expression levels observed in the corresponding ADAM8-expressing control cells (either Panc89_siControl in Fig. 4c or Panc1_A8_siControl in Fig. 4e, red bars). These results suggest that NEAT1 might be directly regulated by miRNA-181a-5p, which was further analyzed by a dual luciferase assay (Fig. 4f). Using the online databases DIANA and StarBase, two predicted miR-181a-5p binding sites on NEAT1 were identified (Fig. 4g). To corroborate this, the luciferase reporter construct containing the two potential binding sites for miRNA-181a-5p in the NEAT1_1 transcript at the 3’ end of the luciferase encoding region (Fig. 4f) was constructed with either the wild-type (WT bsA and B) or the mutated (MUT bsA and B) sequence. These constructs were transfected into wild-typePanc89 cells (Panc89_WT), and the luciferase activities were determined by dual luciferase assays. Both wild-type but not mutated binding sites resulted in a marked decrease in luciferase activity after mimic miRNA-181a-5p expression (Fig. 4h). These data suggest that miRNA-181a-5p can directly suppress NEAT1_1 transcription.

### NEAT1 interacts with STAT3 in PDAC cells

**Fig. 5:**
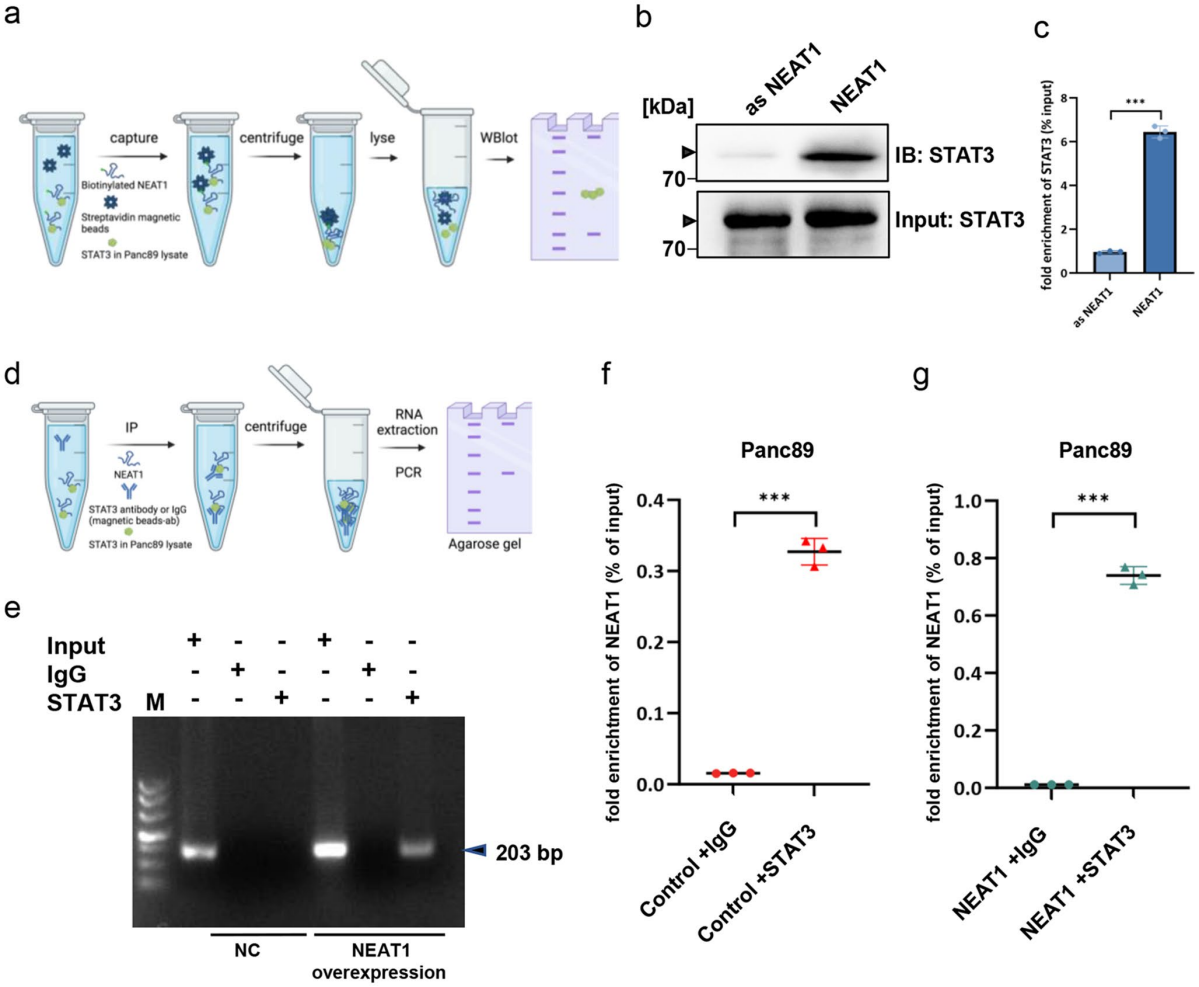
NEAT1 interacts with STAT3 in PDAC cells. **a** Scheme of the pull-down assay using biotinylated NEAT1, antisense NEAT1 as a negative control, streptavidin, and cell lysate from Panc89 cells. **b** After centrifugation, western blotting was performed to detect either STAT3 (input: STAT3) or STAT3 pulled down by biotin/streptavidin (IB: STAT3) and sense NEAT1 (NEAT1) but not by the antisense (as NEAT1) NEAT1 (*n* = 3). **c** Quantitative results of (**b**) from 3 independent experiments. **d** Schematic of the experimental setup for the RNA immunoprecipitation assay with either an unrelated IgG (negative control) or STAT3 antibody and subsequent PCR detection (**e**) and RT-qPCR of NEAT1 in either Panc89 cells (**f**) or Panc89 cells overexpressing NEAT1 (**g**) (*n* = 3). The NEAT1 sequence precipitated with the STAT3 antibody was detected by PCR, which revealed the potential binding site of STAT3 to NEAT1, resulting in a 203 bp fragment in a 1.5% agarose gel. The data are presented as the mean ± S.D and Student’s *t test* was used with ***p < 0.001

According to a previous report on CD4^+^ T cells, NEAT1 can stabilize STAT3 protein levels by direct binding, potentially reducing STAT3 degradation [24]. We hypothesized that the ADAM8-dependent effect on aberrant STAT3 signaling in PDAC cells could be based on a similar mechanism. Clinical data from the Marburg patient cohort revealed a positive correlation between NEAT1 and STAT3 expression at the RNA level (*r* = 0.749, *p* = 2.5×10^−10^, data not shown). Binding of NEAT1 to STAT3 could be instrumental for the function of NEAT1 in STAT3 signaling. To test this possibility, two methods were used to provide evidence for an interaction of NEAT1 with STAT3: (i) a pull-down assay using biotinylated NEAT1 (either antisense NEAT1 or sense NEAT1) and lysates from Panc89_WT cells. After streptavidin capture, the lysates were analyzed for STAT3 by Western blotting and quantified (Fig. 5b and c). The NEAT1 sense sequence, but not the antisense NEAT1 sequence, was shown to be associated with the STAT3 protein. (ii) IP using STAT3 or a related IgG was used to pull down STAT3 from lysates of either Panc89_WT or Panc89 cells overexpressing NEAT1. Subsequent PCR and RT-qPCR analysis revealed that in both cell lysates, NEAT1 was only enriched in samples immunoprecipitated with the STAT3 antibody but not with a related IgG in Panc89_WT cell lysates expressing NEAT1 (Fig. 5d–g). Both results support the notion that there might be an interaction between the lncRNA NEAT1 and the STAT3 protein that could affect STAT3 degradation.

### NEAT1 regulates STAT3 signaling in an ADAM8-dependent manner

**Fig. 6:**
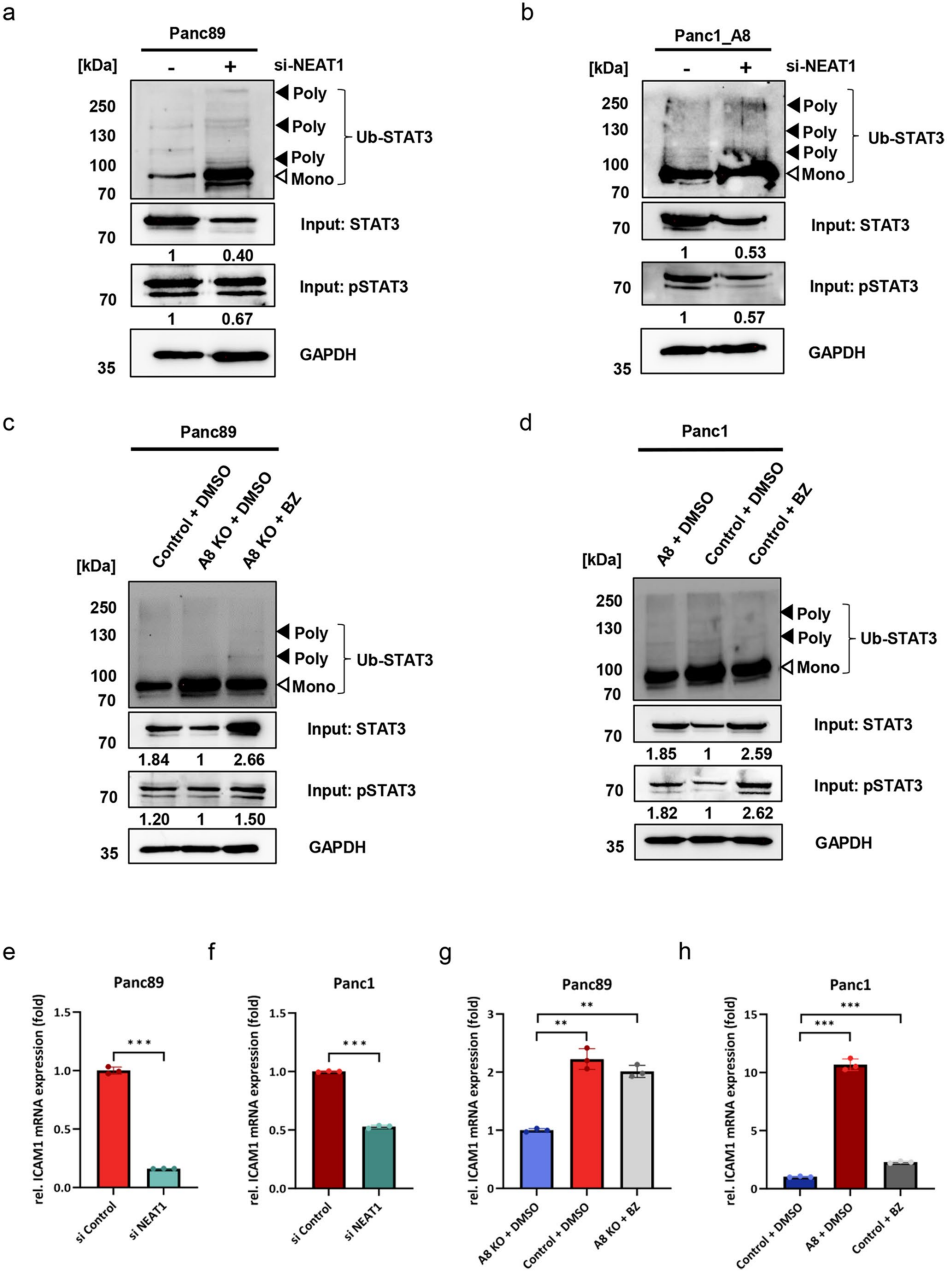
NEAT1 regulates STAT3 signaling in an ADAM8-dependent manner. **a** siRNA-mediated knockdown of NEAT1 in Panc89 cells and western blot analysis of STAT3 ubiquitination. The levels of total STAT3, pSTAT3, and GAPDH, which were used as loading controls, are shown below. **b** Similar to (**a**), NEAT1 was knocked down in Panc1_A8 cells, which increased the level of ubiquitinated STAT3, concomitant with decreased levels of total STAT3 and pSTAT3. GAPDH served as a loading control. **c** Detection of ubiquitinated STAT3 levels in Panc89_WT, Panc89_A8KO and botezomib treated Panc89_A8KO cells by Western blot. Notably, the ubiquitinated STAT3 levels in Panc89_WT and botezomib treated Panc89_A8KO cells were higher than those in Panc89_A8KO cells; compared with those in Panc89_A8KO cells, the total STAT3 and pSTAT3 levels in the Panc89_WT and Panc89_A8KO cells treated with bortezomib (1 ng/mL) for 16 hours increased. **d** Compared with those in Panc1_WT cells, ADAM8 overexpression in Panc1 cells or treatment with bortezomib (1 ng/mL) for 16 hours decreased the levels of ubiquitinated STAT3 while increased total STAT3 and pSTAT3 levels. **e** As a result of NEAT1 knockdown in Panc89_WT cells, the expression of potential target genes of STAT3, such as ICAM1, was analyzed by RT-qPCR. **f** NEAT1 knockdown inhibited ICAM1 mRNA expression in Panc1_A8 cells. **g** ADAM8 knockout inhibited ICAM1 mRNA expression in Panc89_A8KO cells, and treatment with bortezomib (1 ng/mL) for 16 hours restored ICAM1 mRNA expression in Panc89_A8KO cells. **h** ADAM8 overexpression promoted ICAM1 mRNA expression in Panc1 cells, and treatment with bortezomib (1 ng/mL) for 16 hours restored ICAM1 mRNA expression in Panc1 cells. Data are presented as mean ± S.D and as statistical test, Mann-Whitney and Kruskal-Wallis tests were used with *p < 0.05; **p < 0.01; ***p < 0.001

**Fig. 7:**
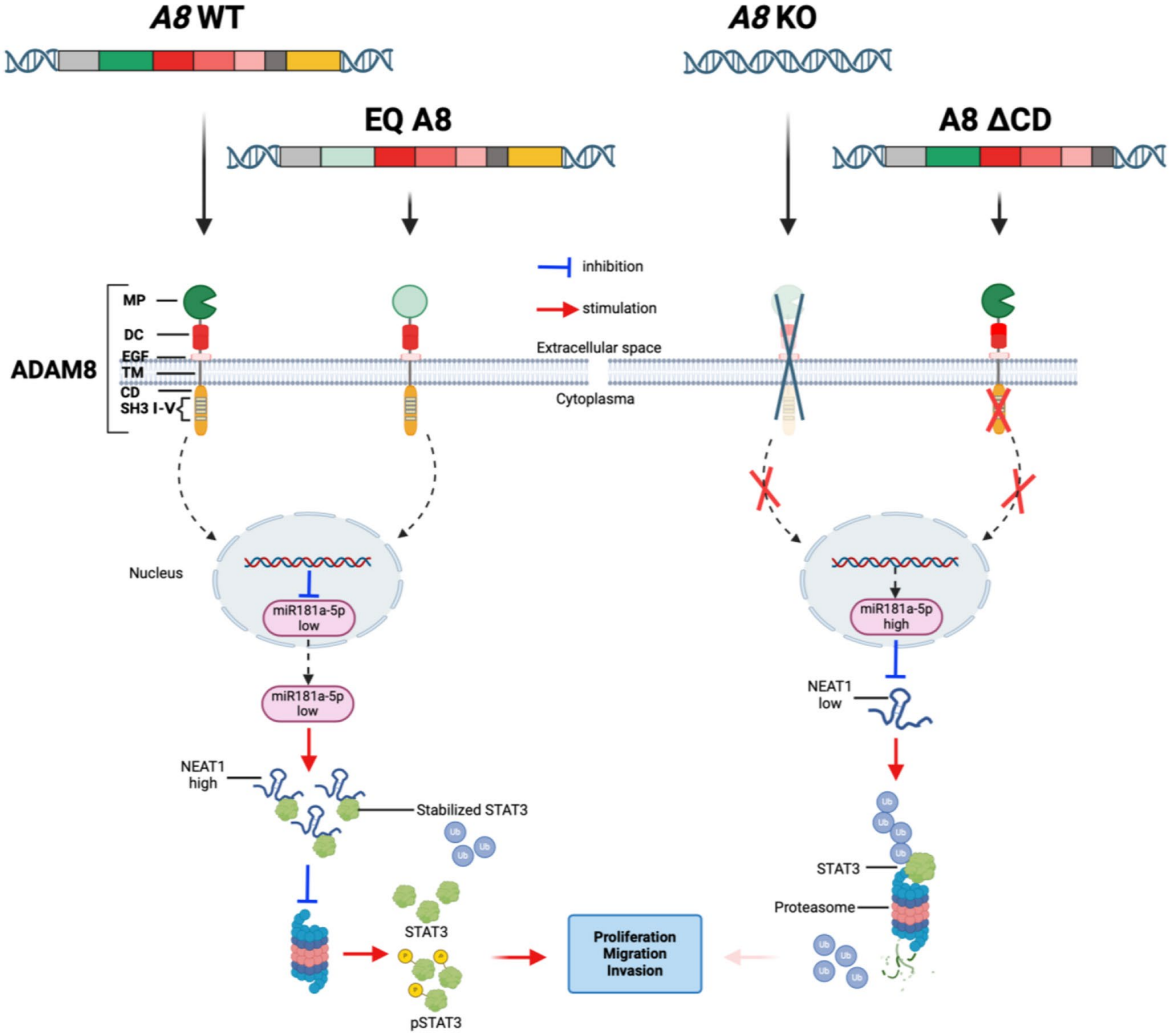
Graphical sketch of how ADAM8 affects STAT3 levels via the regulation of miR-181a-5p and NEAT1. In the left half, either wild-type or protease-deficient full-length ADAM8 can suppress miR-181a-5p expression in PDAC cells, leading to high expression levels of NEAT1 in the cytoplasm. NEAT1 binds to STAT3, and this complex is not degraded in the proteasome; thus, STAT3 signaling remains high in ADAM8-expressing cells (red arrow below) to promote the downstream effects of STAT3, such as proliferation, migration, and invasion. The right half shows the situation in which ADAM8 is either deficient or the cytoplasmic domain is missing. This leads to high levels of miR-181a-5p that suppress NEAT1 expression and ubiquitination of STAT3 with subsequent degradation in the proteasome, thereby downregulating STAT3 signaling in PDAC cells. Abbreviations: A8/ADAM8, A disintegrin and metalloproteinase 8; MP, metalloprotease; DC, a disintegrin-like and a cysteine-rich; EGF, epidermal growth factor-like; TM, transmembrane; CD, cytoplasmic tail; SH3, Src homolog 3; WT, wild type; KO, knockout; EQ, the protease-inactive variant ADAM8 with amino acid exchange E to Q at aa position 335; DCD, an ADAM8 construct lacking the cytoplasmic domain; STAT3, signal transducer and activator transcription 3; p-STAT3: phosphorylated STAT3; NEAT1: nuclear enriched abundant transcript 1

Next we investigated whether the supposed interaction between NEAT1 and STAT3 could directly affect STAT3 degradation. To address this, we used Panc89_WT and Panc1_A8 cells, both of which express physiological levels of ADAM8, and subjected these cells to a ubiquitination assay in the presence or absence of NEAT1. After an efficient knockdown of NEAT1 (decreased to 0.2 compared to siRNA control; Fig. S3a and d), the amount of ubiquitinated STAT3 increased significantly in both cell lines (Figs. 6a, b, and S3), as seen by a larger amount of mono-ubiquitinated STAT3 and some larger aggregates containing poly-ubiquitinated STAT3 protein (Fig. 6a and b). Concomitantly, the amount of STAT3/p-STAT3 in the lysate decreased to 0.4 for STAT3 and 0.67 for p-STAT3 in Panc89_WT cells, and to 0.53 for STAT3 and 0.57 for p-STAT3 in Panc1_A8 cells. When comparing Panc89 control with A8KO cells, a more prominent ubiquitination was observed in A8KO cells, in which the level of STAT3 decreased from 1.84 to 1 and the level of p-STAT3 decreased from 1.2 to 1, indicating that increased ubiquitination could lead to increased proteasomal degradation since less protein was detected. This effect was further verified using bortezomib, a potent proteasome inhibitor, at a sublethal dose of 1 ng/mL (IC_50_ concentrations for Panc1: > 100 ng/mL, for Panc89_A8KO: 25 ng/mL). In Panc89_A8KO cells, treated with bortezomib, STAT3 levels were increased to those in control cells without bortezomib treatment (1 to 2.66 for STAT3 and 1 to 1.5 for p-STAT3; Fig. 6c) which indicates that preventing ubiquitination and subsequent degradation in the proteasome has a profound effect on STAT3 signaling. Similar results with bortezomib were obtained for Panc1_A8 cells; in Panc1 control cells treated with bortezomib, STAT3 levels increased from 1 to 2.59, and p-STAT3 levels increased from 1 to 2.62, higher than those in Panc1_A8 cells without bortezomib treatment (1.85 for STAT3 and 1.82 for p-STAT3; Fig. 6d). We further tested whether STAT3 signaling altered by ADAM8 and bortezomib directly affects gene regulation. To this end, we quantified the expression levels of a representative STAT3-dependent gene, *ICAM-1* (Fig. 6e–h). Expression changes of the *ICAM-1* gene correlated well with the ubiquitination status of STAT3 in Panc89_WT and Panc1_A8 cells (Fig. 6e and f) after NEAT1 knockdown. *ICAM-1* expression in bortezomib treated Panc89_A8KO and Panc1_WT cells respectively, is similar to the expression in Panc89_WT cells and, to a lesser extent, to the expression in Panc1_A8 cells. We conclude that the extent to which STAT3 is prevented from being degraded by NEAT1 reflects the signaling capacity of STAT3 and could be therefore of high pathophysiological importance. A graphical summary of the results is shown in Fig. 7.

## Discussion

The importance of STAT3 in inflammation and PDAC progression has been demonstrated in many studies. STAT3 signaling is activated by the binding of various ligands to their surface receptors, leading to phosphorylation of STAT3. STAT3 can be also directly phosphorylated by non-receptor tyrosine kinases, Src and Abl. Phosphorylated STAT3 is further homo-dimerized and the phosphorylation of tyrosine 705 residue is responsible for nuclear translocation of STAT3 [27]. Our study adds another level of STAT3 activity regulation in tumor cells, namely, the regulation of STAT3 stability/degradation. There are a total of 26 ubiquitination sites in the STAT3 protein, as deduced from the PhosphoSitePlus database (http://www.Phosphosite.org; access date 04/22/2024). Six of these sites are located in the DNA binding domain, including lysine residues K348, K363, K365, K370, K383, and K409. In addition, K177, K244, and K294 are ubiquitination sites at the all-alpha domain of STAT3, whereas K626 is located in the SH2 dimerization domain of STAT3. From the crystal structure of STAT3, it is likely that one particular lysine residue, K348, important for DNA binding could be sterically blocked by NEAT1 binding thereby preventing STAT3 degradation. As the NEAT1 sequence contains canonical STAT3 DNA binding sites, the physical interaction between the STAT3 protein and NEAT1 could compete with DNA binding of STAT3 and thereby reducing STAT3 signaling. With regard to E3 ligases, there are two known E3 ligases, COP1 and MDM2 (similar to p53) binding to STAT3, and there are 88 additional predicted E3 ligases for STAT3. The top two predicted E3 ligases with high confidence were WWP2 and NEDD4L (source: UbiBrowser 2.0). In future work, this complex network needs to be untangled to fully understand the regulation of STAT3 by ubiquitination in PDAC.

Although ADAM8 has been validated as a clinically relevant drug target, its precise role in PDAC has still not been fully characterized, as binding to integrin β1 and/or proteolytic activity alone cannot account for the observed significant effects on PDAC progression [8]. Here we revealed, for the first time to our knowledge, an intracellular pathway in which ADAM8, a metalloprotease-disintegrin that is clearly distinct from the classical membrane shedding enzyme, regulates aberrant STAT3 signaling by controlling the expression of the intracellular lncRNA NEAT1 in pancreatic cancer cell lines via ADAM8-dependent miR-181a-5p expression. Based on numerous previous studies, ADAM8 plays a crucial role in promoting tumor cell migration and invasion in various cancers [28–30], and the profound effect of its cytoplasmic domain on intracellular signaling in PDAC is demonstrated here. The aberrant expression of miRNAs in cancers has been reported in various studies, and miR-181a-5p, a tumor suppressor in some tumor entities, has also been described [31]. Our study revealed a negative correlation between ADAM8 expression and miR-181a-5p levels in PDAC cell lines. Given this correlation, the effects of ADAM8 deficiency or miR-181a-5p overexpression on tumor suppression were similar. Mechanistically, the regulatory effect of ADAM8 on miR-181a-5p expression is mainly due to its cytoplasmic domain which contains five SH3 domains. As previous research indicated, ADAM8 could bind to integrin β1 in PDAC. But miR-181a-5p expression was not affected in either Panc89 or Panc1_A8 cells after integrin β1 knockdown in this study (Fig. S4). In contrast, using an SH3 inhibitor could successfully reverse the ADAM8 mediated repression of miR-181a-5p. To refine the search for signaling pathways, we corroborated the effect of several pathway inhibitors on the suppression of miRNA 181a-5p expression by ADAM8. Notably, by using Dactolisib (10 mM) in Panc89 cells, we identified the mTOR pathway as the most relevant signaling to mediate miRNA181a-5p and downstream NEAT1 expression (Gao and Bartsch, unpublished data). Human cancers are associated with altered transcription patterns of lncRNAs and/or miRNAs [32]. In recent years, increasing knowledge has been gathered on the occurrence and complexity of RNA-mediated regulatory circuitries, including RNA‒RNA interactions, especially interactions between miRNAs and lncRNAs via complementary base pairing [33]. According to the GEPIA2 and TCGA databases, NEAT1 is highly expressed in pancreatic cancer and is positively correlated with ADAM8. The NEAT1 isoform of the short NEAT1_1 transcript of 3.7 kb is more abundant in the cytoplasm and is often associated with diseases [34, 35]. The results of our study demonstrated that cytoplasmic NEAT1_1 was downregulated after ADAM8 knockout in PDAC cells and that the cytoplasmic domain of ADAM8 plays an essential role in regulating NEAT1 levels. NEAT1 knockdown in PDAC cells has the same anti-tumoral effects as ADAM8 deficiency and miR-181a-5p overexpression: reduced cell proliferation and inhibited cell migration and invasion (Fig. S6). In addition, bioinformatic analysis suggested that the lncRNA NEAT1 is a target of miR-181a-5p, and two binding sites of miR-181a-5p in NEAT1 with high scores were functionally identified. Through gain- and loss-of-function assays and dual luciferase reporter gene assays, we revealed that the tumor suppressive effects of miR-181a-5p are partially attributed to its downregulation of NEAT1.

STAT3 is an important transcription factor in tumor progression, and its activation is pivotal in regulating the expression of EMT-related genes/proteins, such as Twist, Vimentin, Snail, MMPs, and ZEB1, essential proteins in metastasis [36]. Our present study revealed that the STAT3 signaling pathway is partially regulated by ADAM8 in pancreatic cancer, but only the STAT3 protein, not its mRNA, is dependent on ADAM8 expression. The results of the gain- and loss-of-function assays for miR-181a-5p and NEAT1 were also consistent with these results: both NEAT1 knockdown and miR-181a-5p overexpression decreased STAT3 protein but not STAT3 mRNA levels. Moreover, the cytoplasmic domain of ADAM8 has a more pronounced effect on STAT3 regulation than its metalloprotease domain. A direct effect of Src kinase in this signaling could not be demonstrated as the Src inhibitor PP2 was not efficient to impede miR-181a-5p suppression in Panc89_WT and Panc1_A8 cells. In contrast, using an inhibitor that was shown to be effective for blocking binding of kinases lyn and fyn to the SH3 domain could successfully reverse the ADAM8 mediated repression of miR-181a-5p, supporting the notion that these kinases may be important for miR-181a-5p regulation.

Increasing evidence was provided that NEAT1 is overexpressed in many solid tumors, and higher expression of NEAT1 is correlated with poor survival, recurrence and tumor metastasis [37–39]. Mechanistically, in addition to sponging some miRNAs and stabilizing certain mRNAs, the secondary structure of NEAT1 can capture some proteins and change their stability or molecular function [40]. The functions of NEAT1, together with some specific proteins that form complexes, can be divided into three types: guide, decoy and scaffold [41]. In addition, transcription factors include NEAT1-binding proteins [24]. Our results showed that NEAT1 targets the STAT3 protein via physical interaction and inhibition of its ubiquitination. These results have expanded the knowledge about the importance of ADAM8 in pancreatic cancer and shed light on the molecular mechanisms by which the ADAM8/miR-181a-5p/NEAT1 signaling axis regulates the STAT3 signaling pathway. We conclude that this pathway could be relevant for PDAC pathology, and reveal new perspectives for the therapeutic application of miRNA therapy options in the future, for example by targeted delivery of miRNA181a-5p in PDAC.

## Electronic supplementary material

Below is the link to the electronic supplementary material.

Supplementary Material 1

Supplementary Material 2

## Data Availability

All data presented here can be made available upon reasonable request.

## Abbreviations

PDAC: Pancreatic ductal adenocarcinoma
ADAM8: A disintegrin and metalloproteinase 8
STAT3: Signal transducer and activator of transcription 3
p-STAT3: phosphorylated STAT3
lncRNA: Long noncoding RNA
NEAT1: Nuclear enriched abundant transcript 1
miRNA: microRNA
TM: Transmembrane
DIS: Disintegrin
EGF: Epidermal growth factor
FAK: Focal adhesion kinase
ERK: Extracellular regulated kinase
MAPK: Mitogen-activated protein kinase
MEN1: Multiple endocrine neoplasia locus1
TCGA: The Cancer Genome Atlas
KO: knockout
SH3: SRC Homology 3
WT: wild-type
MUT: Mutated
bs: Binding site
ICAM-1: Intercellular Adhesion Molecule 1
JAK: Janus kinase
GEPIA2: Gene Expression Profiling Interactive Analysis 2
EMT: Epithelial-to-mensenchymal transition
ZEB1: Zinc finger E-box-binding homeobox 1
FBS: Fetal Bovine Serum
RT-qPCR: Reverse transcription-quantitative polymerase chain reaction
RIP: RNA binding protein immunoprecipitation
RBP: RNA binding protein
VRC: Vanadyl ribonucleoside complex
PCR: Polymerase chain reaction
CT: Control
IB: Immunoblotting
as: Antisense
siRNA: Small interfering RNA
DMSO: Dimethyl sulfoxide
IS: Immunoscore
TPM: Transcripts per million
