# Supplementary material for: The long non-coding RNA NEAT1 contributes to aberrant STAT3 signaling in pancreatic cancer and is regulated by a metalloprotease-disintegrin ADAM8/miR-181a-5p axis
Source: Cell Oncol (Dordr). 2024 Oct 16;48(2):391–409. doi: 10.1007/s13402-024-01001-0 (PMC11996950; doi:10.1007/s13402-024-01001-0)
Supplement: Supplementary file 1 — Supplementary Material 1 [file 13402_2024_1001_MOESM1_ESM.docx]

**Legends to Supplementary Figures:**

**Fig. S1** STAT3 and pSTAT3 levels were positively correlated with ADAM8 levels in PDAC cells. (**a**)-(**d**) Lumit immunoassay results showing STAT3 and pSTAT3 levels in Panc1 and Panc89 cells, respectively. The data are presented as the mean ± S.D. Each experiment was performed once in triplicate.

**Fig. S2** NEAT1_2 expression in the cytoplasm and nucleus was not regulated by ADAM8 in PDAC cells. (**a**) Cytoplasmic NEAT1_2 expression in Panc1_WT cells and Panc1_A8 cells. (**b**) Cytoplasmic NEAT1_2 expression in Panc89_WT cells and Panc89_A8 ko cells. (**c**) Nuclear NEAT1_2 expression in Panc1_WT cells and Panc1_A8 cells. (**d**) Nuclear NEAT1_2 expression in Panc89_WT cells and Panc89_A8 ko cells. The data are presented as the mean ± SD and Student’s *t test* was used to determine statistical significance (**p*<0.05; ***p<*0.01; ****p*<0.001)*.*

**Fig. S3** NEAT1 regulates STAT3 levels in an ADAM8-dependent manner. (**a**) NEAT1 knockdown in Panc89_WT cells. (**b**) and (**c**) Quantification of the Western blotting results shown in Figure 6A. (**d**) NEAT1 knockdown in Panc1_A8 cells. (**e**) and (**f**) Quantification of the Western blotting results shown in Figure 6B. (**g**) and (**h**) Quantification of the Western blotting results shown in Figure 6C. (**i**) and (**j**) Quantification of the Western blotting results shown in **Fig. 6d**. Each experiment was performed once.

**Fig. S4** Integrin β1 knockdown did not affect miR-181a-5p, NEAT1 or STAT3 expression in PDAC cells. (**a**) Integrin β1 mRNA and protein levels in Panc1 and Panc1_A8 cells. (**b**) STAT3 and pSTAT3 protein expression after integrin β1 knockdown in Panc1_A8 cells. (**c**) Integrin β1 mRNA expression after integrin β1 knockdown in Panc1_A8 cells. (**d**) STAT3 mRNA expression after integrin β1 knockdown in Panc1_A8 cells. (**e**) NEAT1 expression after integrin β1 knockdown in Panc1_A8 cells. (**f**) miR-181a-5p expression after integrin β1 knockdown in Panc1_A8 cells. (**g**) Integrin β1 mRNA and protein levels in Panc89_WT and Panc89_A8KO cells. (**h**) STAT3 and pSTAT3 protein expression after integrin β1 knockdown in Panc89_WT cells. (**i**) Integrin β1 mRNA expression after integrin β1 knockdown in Panc89_WT cells. (**j**) STAT3 mRNA expression after integrin β1 knockdown in Panc89_WT cells. (**k**) NEAT1 expression after integrin β1 knockdown in Panc89_WT cells. (**l**) miR-181a-5p expression after integrin β1 knockdown in Panc89_WT cells. The data are presented as the mean ± S.D. Each experiment was performed once.

**Fig. S5** An SH3 inhibitor inhibited the expression of miR-181a-5p but not NEAT1 or STAT3 in Panc89_WT cells. (**a**) miR-181a-5p expression in Panc1 cells after treatment with DMSO, 1 μM PP2, 10 μM PP2 or 10 μM SH3 inhibitor (4b). (**b**) NEAT1 expression in Panc1 cells after treatment with DMSO, 1 μM PP2, 10 μM PP2 or 10 μM SH3 inhibitor (4b). (**c**) STAT3 mRNA levels in Panc1 cells after treatment with DMSO, 1 μM PP2, 10 μM PP2 or 10 μM SH3 inhibitor. (**d**) STAT3 and pSTAT3 protein levels in Panc1 cells after treatment with DMSO, 1 μM PP2, 10 μM PP2 or 10 μM SH3 inhibitor. (**e**) miR-181a-5p expression in Panc89_WT cells after treatment with DMSO, 1 μM PP2, 10 μM PP2 or 10 μM SH3 inhibitor. (**f**) NEAT1 expression in Panc89_WT cells after treatment with DMSO, 1 μM PP2, 10 μM PP2 or 10 μM SH3 inhibitor. (**g**) STAT3 mRNA levels in Panc1 cells after treatment with DMSO, 1 μM PP2, 10 μM PP2 or 10 μM SH3 inhibitor. (**h**) STAT3 and pSTAT3 protein levels in Panc1 cells after treatment with DMSO, 1 μM PP2, 10 μM PP2 or 10 μM SH3 inhibitor. The data are presented as the mean ± S.D. Each experiment was performed once.

**Fig. S6** NEAT1 knockdown reduced cell proliferation and inhibited cell migration and invasion in Panc89_WT cells. (**a**) NEAT1 knockdown efficiency in Panc89_WT cells. (**b**) NEAT1 knockdown reduced cell proliferation in Panc89_WT cells. (**c**) NEAT1 knockdown inhibited cell migration in Panc89_WT cells. (**d**) The quantification of (**c**). (**e**) NEAT1 knockdown inhibited cell invasion in Panc89_WT cells. (**f**) The quantification of (**e**). The data are presented as the mean ± S.D (**p*<0.05; ***p<*0.01)*.* Each experiment was performed three times.
