# Supplementary figures and images for: The long non-coding RNA NEAT1 contributes to aberrant STAT3 signaling in pancreatic cancer and is regulated by a metalloprotease-disintegrin ADAM8/miR-181a-5p axis
Source: Cell Oncol (Dordr). 2024 Oct 16;48(2):391–409. doi: 10.1007/s13402-024-01001-0 (PMC11996950; doi:10.1007/s13402-024-01001-0)

Figure S1.

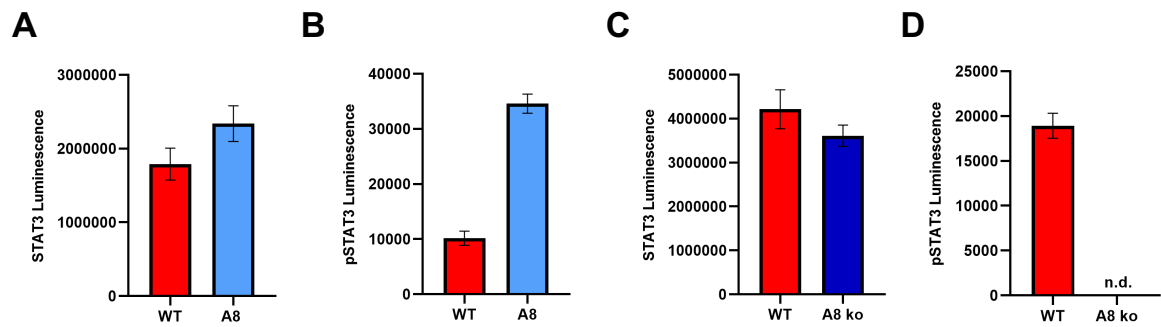

Figure S2.

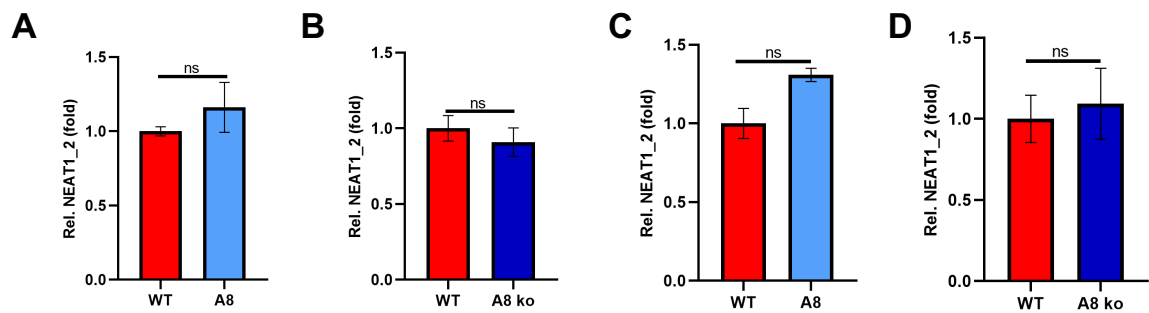

Figure S3.

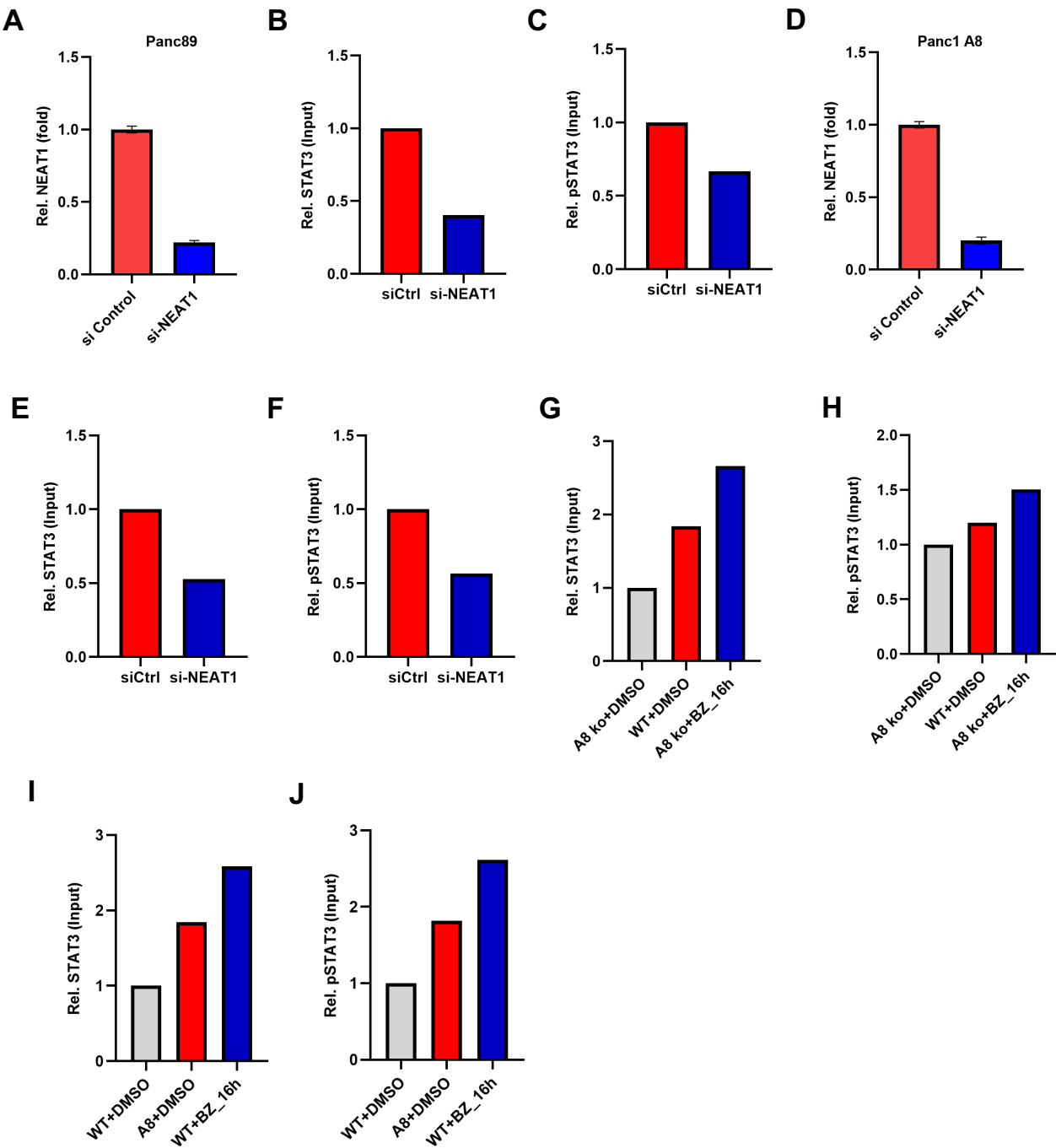

Figure S4.

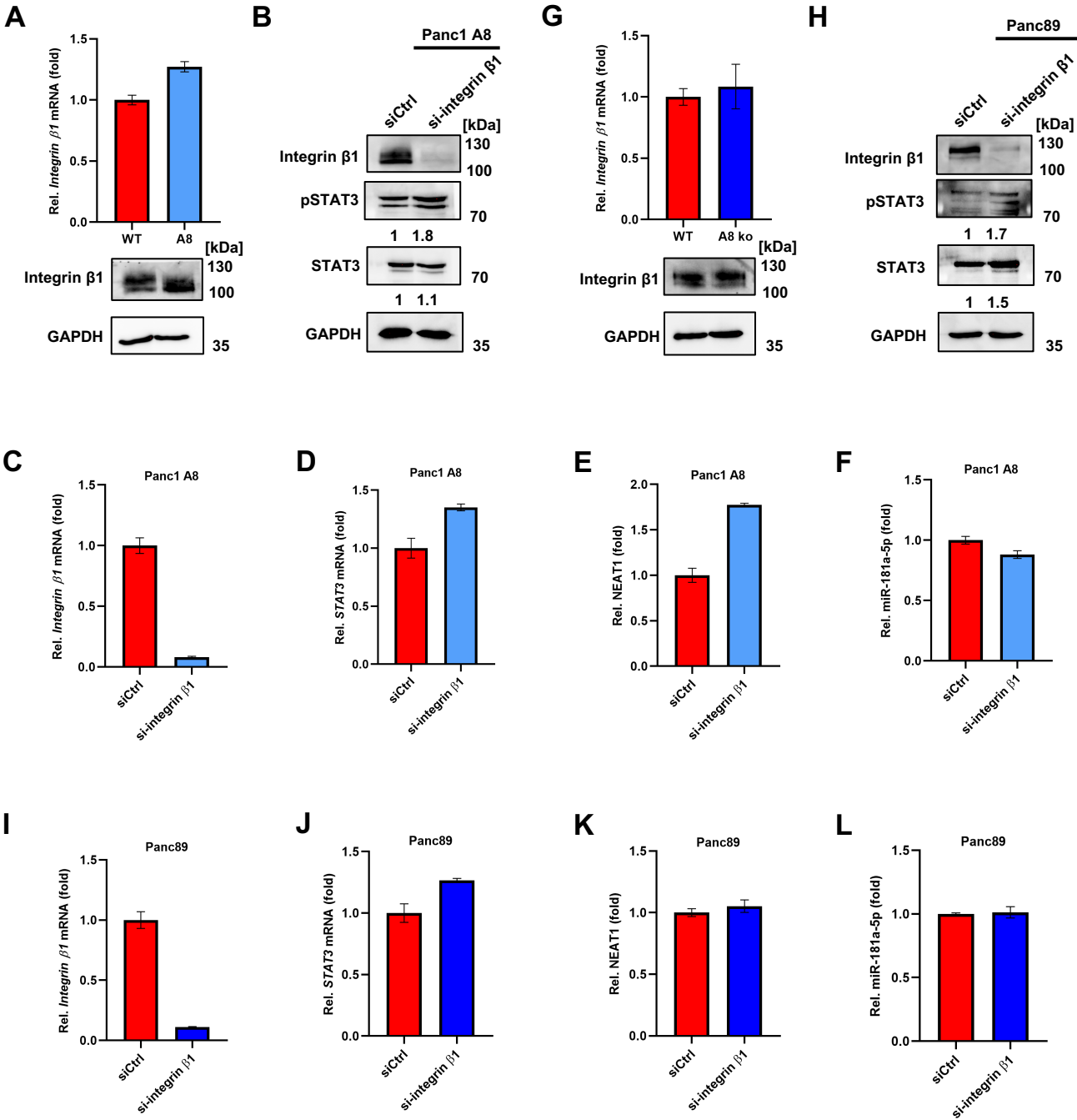

Figure S5.

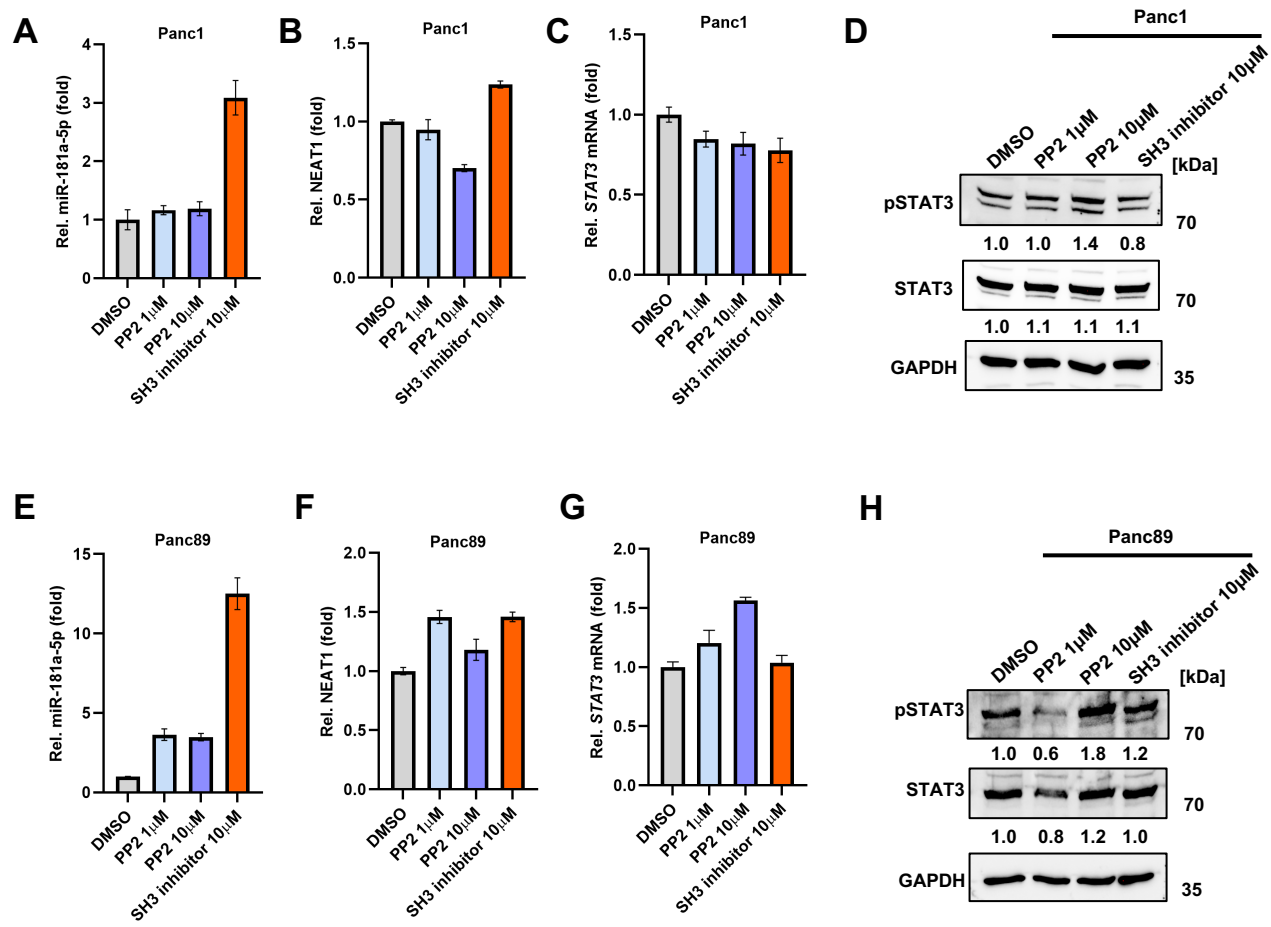

Figure S6.

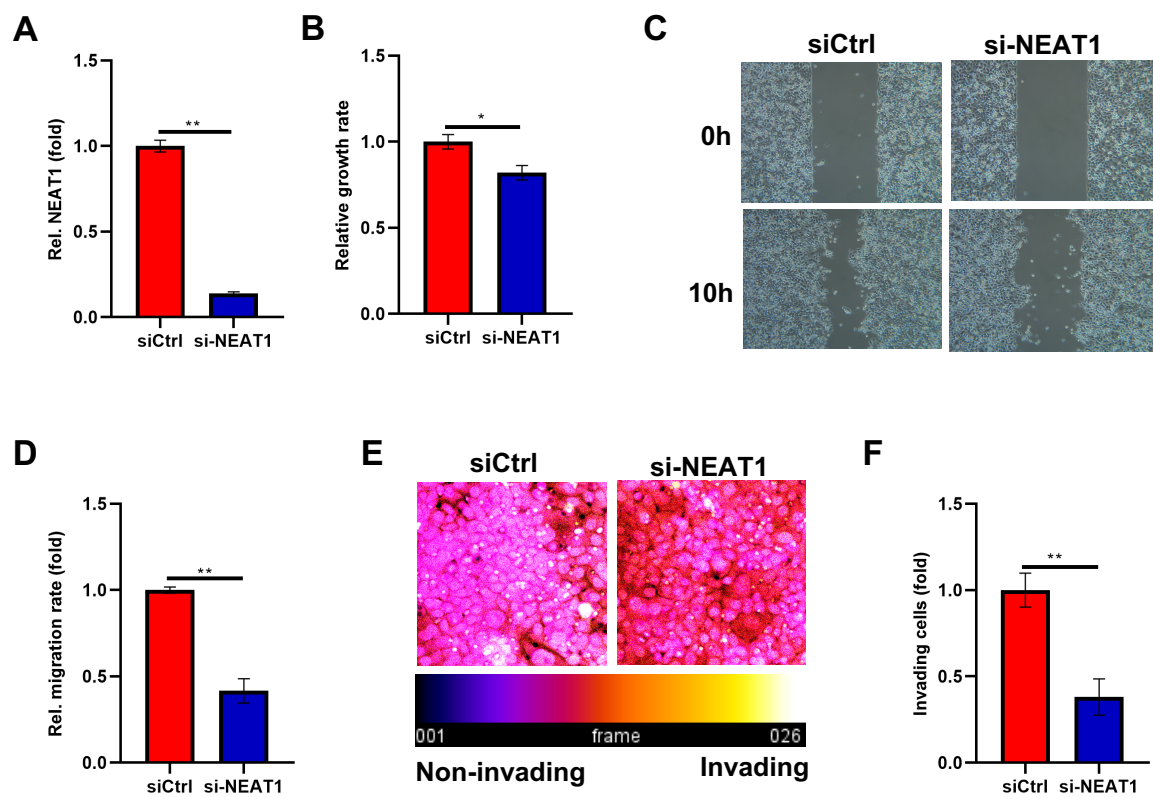

Supplement: Supplementary file 2 — Supplementary Material 2 [file 13402_2024_1001_MOESM2_ESM.pdf]
